# Comparative Analysis of Dorsal Root, Nodose and Sympathetic Ganglia for the Development of New Analgesics

**DOI:** 10.3389/fnins.2020.615362

**Published:** 2020-12-23

**Authors:** Matthew R. Sapio, Fernando A. Vazquez, Amelia J. Loydpierson, Dragan Maric, Jenny J. Kim, Danielle M. LaPaglia, Henry L. Puhl, Van B. Lu, Stephen R. Ikeda, Andrew J. Mannes, Michael J. Iadarola

**Affiliations:** ^1^Anesthesia Section, Department of Perioperative Medicine, National Institutes of Health Clinical Center, Bethesda, MD, United States; ^2^Flow and Imaging Cytometry Core Facility, National Institute of Neurological Disorders and Stroke, National Institutes of Health, Bethesda, MD, United States; ^3^Section on Neurotransmitter Signaling, National Institute on Alcohol Abuse and Alcoholism, National Institutes of Health, Bethesda, MD, United States

**Keywords:** dorsal horn, intermediolateral cell column (IML), dorsal root ganglia, sympathetic ganglia, nodose ganglia, RNA-Seq, transcriptome, opioid

## Abstract

Interoceptive and exteroceptive signals, and the corresponding coordinated control of internal organs and sensory functions, including pain, are received and orchestrated by multiple neurons within the peripheral, central and autonomic nervous systems. A central aim of the present report is to obtain a molecularly informed basis for analgesic drug development aimed at peripheral rather than central targets. We compare three key peripheral ganglia: nodose, sympathetic (superior cervical), and dorsal root ganglia in the rat, and focus on their molecular composition using next-gen RNA-Seq, as well as their neuroanatomy using immunocytochemistry and *in situ* hybridization. We obtained quantitative and anatomical assessments of transmitters, receptors, enzymes and signaling pathways mediating ganglion-specific functions. Distinct ganglionic patterns of expression were observed spanning ion channels, neurotransmitters, neuropeptides, G-protein coupled receptors (GPCRs), transporters, and biosynthetic enzymes. The relationship between ganglionic transcript levels and the corresponding protein was examined using immunohistochemistry for select, highly expressed, ganglion-specific genes. Transcriptomic analyses of spinal dorsal horn and intermediolateral cell column (IML), which form the termination of primary afferent neurons and the origin of preganglionic innervation to the SCG, respectively, disclosed pre- and post-ganglionic molecular-level circuits. These multimodal investigations provide insight into autonomic regulation, nodose transcripts related to pain and satiety, and DRG-spinal cord and IML-SCG communication. Multiple neurobiological and pharmacological contexts can be addressed, such as discriminating drug targets and predicting potential side effects, in analgesic drug development efforts directed at the peripheral nervous system.

## Introduction

Negative central nervous system (CNS) side effects of analgesic drugs such as opioids have driven a renewed interest in peripherally directed analgesics. The goal of peripherally acting drugs is to block pain-producing nociceptive sensations while sparing non-painful sensations such as light touch and vibration. This approach entirely avoids CNS effects, circumventing actions such as addiction or respiratory suppression to provide safer pain control. In the development of such compounds, a comprehensive molecular atlas of peripheral nervous system targets is useful for the prediction of both on-target and off-target effects. Predictions of drug action can be greatly informed by determining the presence or absence of a particular drug target on neurons in sensory and/or autonomic peripheral ganglia. For example, molecules located on sensory afferents, but not sympathetic efferents may represent appropriate targets for blockade of sensory activation without sympathetic side effects. Knowing the ganglionic expression pattern can also facilitate design of localized interventional injection approaches to minimize these effects (Iadarola and Gonnella, 2013; Iadarola et al., 2018; Sapio et al., 2018).

Sensory information is transduced by a wide variety of nerve endings that inform the body about what is going on in the outside world and within its organ systems. Sensory information such as touch and pain are transmitted to the spinal cord via the dorsal root (DRG) and trigeminal ganglia (TG), whereas vagal afferents innervate several areas of the viscera and alimentary canal. These vagal afferents have cell bodies in the nodose ganglion (NDG), which projects to the nucleus of the solitary tract or area postrema in the brainstem. In response to these afferent stimuli, sympathetic efferents also become engaged, presumably through spinal circuits routed at the level of the intermediolateral cell column (IML). The superior cervical ganglion (SCG) receives input from the IML and projects to the end organs. These axons are referred to as the post-ganglionic sympathetic efferents because they project from the soma in the SCG. The fibers from the SCG participate in sympathetic control of the iris, the glands and blood vessels of the head and face, and presumably are also representative of those in other ganglia comprising the sympathetic chain.

The present data show that each ganglion is exemplified by a distinct molecular fingerprint constructed using the most highly expressed and most differential genes. These profiles inform the specific functions of each ganglia. Comparisons and contrasts are made between the three ganglia as well as with databases of tissue-level expression to identify transcriptomic signatures of each region. In these analyses, we focus on the specific nociceptive character of the DRG, as we have described previously (Goswami et al., 2014b; Sapio et al., 2016, 2018; Iadarola et al., 2018), and report that the nodose has a similar, albeit exaggerated transcriptomic signature of most nociceptive molecules, indicating the high density of sensory transducing molecules within this ganglion. We suggest that the nodose uses these same transducing molecules to detect noxious stimuli within the viscera, most likely leading to appetite regulation, nausea, and other vagal components of the response to injury, inflammation or chemical insult. These studies present a comprehensive analysis of these three major classes of peripheral ganglia. The use of deep sequencing facilitates ganglionic comparisons and supports the use of such datasets for translational pain research.

## Materials and Methods

### Animal Care and Ethics Approval

Experiments were approved by the Institutional Animal Care and Use Committee of the Clinical Center, National Institutes of Health (Bethesda, MD, United States). Animals were cared for and tested in accordance with ethical guidelines established in the Guide for Care and Use of Laboratory Animals. Male Wistar (Charles River) or Sprague-Dawley rats (Envigo; 200–300 g) were housed in pairs with 12 h light-dark cycles, fed *ad libitum*, and were tested and monitored for behavior during the animal’s light cycle. Animal cages were furnished with a plastic tunnel for enrichment.

### Immunohistochemical Staining

Animals were deeply anesthetized and intracardially perfused with phosphate buffered saline followed by 4% paraformaldehyde as a fixative. Subsequently, superior cervical, nodose, trigeminal and dorsal root ganglia were dissected and immersed in 4% paraformaldehyde overnight. For dorsal root ganglion, the sciatic nerve was exposed by blunt dissection and traced to identify L4, L5, and L6, which were collected bilaterally from rats and pooled for RNA extraction. A different set of the ganglia collected in an identical manner were pooled in paraffin blocks for histological analysis. Note that these DRGs contribute to somatotopic innervation of the hind limb, with L4 innervating the lateral hindlimb as well as the dorsal and plantar aspect of the paw. L5 afferents innervate the posterior hindlimb and lateral paw, and L6 innervates the posterior hindlimb as well as parts of the scrotum (in male rats) and the perineum (Takahashi et al., 1995). Fixed ganglionic samples were embedded in paraffin blocks by Histoserv Inc. (Germantown, MD, United States) such that several ganglia of each type were in a single block together. These blocks were sectioned at 6 μm. Paraffin sections of DRG, NDG, and SCG were warmed at 60°C for 20 min, deparaffinized in xylenes three times for 5 min each, and hydrated in a decreasing EtOH gradient for approximately 1 min each. Antigen retrieval was performed using Citrate Buffer (10 mM Citric Acid, 0.05% Tween-20, pH 6.0) in a 1200 W microwave for 3 min at 100% power and 5 min at 30% power. Slides were washed in buffer containing 145 mM NaCl, 5 mM KCl, 1.8 mM CaCl_2_, 0.8 mM MgCl_2_, and 10 mM HEPES. Slides were incubated in goat blocking serum (VECTASTAIN Elite ABC HRP Kit (Peroxidase, Rabbit IgG), Vector Labs, Burlingame, CA, United States) for 30 min. The slides were incubated in primary antibody (CGRP, 1:10,000, Peninsula Labs, San Carlos, CA, United States or from M. Iadarola (Traub et al., 1989); OPRM1, 1:1,000, R&D Systems, Minneapolis, MN; TH, 1:2,000 (DRG, SCG) or 1:5,000 (NDG), Invitrogen, Carlsbad, CA, United States) diluted in antibody diluent (1% BSA, 0.05% sodium azide, and 0.1% Tween-20 in PBS). The slides were washed in buffer for 5 min and incubated in biotinylated secondary antibody from the Vectastain Kit for 30 min. The slides were washed again for 5 min in buffer and incubated in the Vector ABC Reagent for 30 min. The slides were washed for 5 min in buffer and developed with the ImmPACT DAB kit (Vector, Carlsbad, CA, United States) until optimal color developed [CGRP86, 2 min; CGRP87, 1 min; OPRM1 (DRG), 5 min; OPRM1 (NDG, SCG), 2 min; TH, 1 min]. The slides were rinsed in tap water and counterstained with Hematoxylin (1:10,000, Sigma Aldrich, St. Louis, MO, United States) for 15 s and rinsed in tap water again. The slides were dehydrated in an increasing EtOH gradient for 1 min each, allowed to air dry, cleared three times with xylenes, and again allowed to air dry. The slides were coverslipped using Permount (Fisher Scientific, Waltham, MA, United States). Photomicrographs of slides were captured using an Olympus DP80 camera on an Olympus BX60 microscope or scanned using a Hamamatsu NanoZoomer 2.0HT Digital Slide Scanner (Hamamatsu Photonics, Naka-ku, Hamamatsu). Data were obtained from multiple sections from each rat. More sections (11 sections OPRM1; 20 sections CGRP) were stained to carefully assess a negative result, such as when little or no immunoreactivity was detected for a particular antigen/ganglion combination.

### Multiplex Fluorescent *in situ* Hybridization

For multiplex fluorescent labeling and imaging, methods were performed as described previously with modifications as described subsequently (Sapio et al., 2020). Tissue sections were prepared as described for immunohistochemical staining. Each paraffin block contained several ganglia from all four ganglionic types (DRG, NDG, SCG, and TG) and were stained and scanned simultaneously. After deparaffinization, *in situ* hybridization was performed with RNAscope^®^ Multiplex Fluorescent assays v2.0 (Advanced Cell Diagnostics, Newark, CA, United States) with Tyramide Signal Amplification using 4 Opal dyes (Opal 520, Opal 570, Opal 620, and Opal 690; Perkin Elmer, Waltham, MA, United States). Stained sections were imaged using the Axio Imager.Z2 slide scanning fluorescence microscope (Zeiss, Oberkochen, Germany). Images were captured at 0.325 micron/pixel spatial resolution in 600 μm^2^ tiles. Subsequently, tiles were stitched using ZEN 2 software (Zeiss). Pseudocolored stitched images were overlaid to create multicolored merged composites, and visualized in Adobe Photoshop (San Jose, CA, United States). Additional image analysis was performed using Fiji image analysis software (v 1.0). All probes used in these staining experiments were purchased from Advanced Cell Diagnostics (Bio-Techne, Minneapolis, MN, United States), and the catalog numbers are shown in Supplementary Table 1. Color balancing was considered in the design of the staining combinations, however, some bleed in signal was observed, particularly between the 570 and 620 fluorophores during microscopic image capture. This was generally corrected by subtracting the 570 channel from the 620 channel using Fiji. In all analyses, only signal specific to a single channel was considered.

For quantitation of Figure 2F, images were processed in Fiji (Schindelin et al., 2012) and regions of interest were selected around each cell soma using brightfield and DAPI. Subsequently, intensity of each of the four fluorophores was measured using the “measure” tool. For *Trpv1* and *Trpa1*, a large dynamic range of signal was observed, consistent with previous observations (Sapio et al., 2018). Signal was measured on non-neuronal areas with no specific signal (generally very low) and used as a baseline measurement. Signal below this measurement was not considered above background in the analysis in Figure 2E.

For visualizations of expression overlap in Figures 2D–F, 6, cells were manually examined for positivity of each of the four labels. Counts were tabulated as a binary outcome (positive or negative) for each of the four labels. To create diagrams detailing categorical expression profiling (Figure 6), categories with more than 10 counts were counted as a fraction of total cells. Categories with less than 10 counts were not plotted. Note that cells that did not react with any of the 4 labels were identified using brightfield imaging and DAPI staining, and were plotted in all cases regardless of count number (≤10 counts in Figure 6F). This matrix of four binary variables (one for each channel in the 4-plex image) was imported into R and visualized using the UpSetR package (Conway et al., 2017). The plots generated by UpSetR (Figure 6) show the number of counts for each label individually (*y*-axis) and the counts in each intersection (i.e., combination of labels) on the top of the *x*-axis. This presentation of data is an alternative to high-plex Venn diagrams.

### RNA Extraction, Library Preparation, and Next-Generation Sequencing

Frozen tissue was homogenized in 1 mL of Qiazol (Qiagen, Hilden, Germany) in Lysing matrix D, which contains 1.4 mm ceramic beads, using a Fast Prep-24 Homogenizer (MP Biomedicals) for 20 s at 4.5 m/s (LaPaglia et al., 2018; Sapio et al., 2019). The homogenate was incubated at room temperature for 5 min and was subsequently extracted following the protocol from the RNeasy Lipid Tissue Mini Kit with DNase digestion (Qiagen). RNA was eluted off the column in 50 μL of RNase-free water. RNA quantity and integrity were evaluated using a 2100 Bioanalyzer and the RNA 6000 Nano Kit (Agilent Technologies, Santa Clara, CA, United States). PolyA^+^ mRNA isolation, size fragmentation, cDNA synthesis, size selection, and next gen sequencing were performed at the NIH Intramural Sequencing Center (Rockville, MD, United States) according to their standard protocols, as described previously (Goswami et al., 2014a; LaPaglia et al., 2018; Raithel et al., 2018; Sapio et al., 2018). Briefly, non-stranded cDNA libraries were prepared using the Illumina TruSeq RNA Library Preparation Kit v2 (San Diego, CA, United States) with Biomek (Beckman Coulter, Pasadena, CA, United States) liquid handling automation. The resulting cDNA was fragmented using a Covaris E210 instrument (Woburn, MA, United States). Library amplification was performed using 10 cycles to minimize over-amplification. Unique barcode adapters were applied to each library. Libraries were pooled in equimolar ratio and sequenced together on a HiSeq 2500 with ver 4 flow cells and sequencing reagents. Paired end 125-base read pairs were generated for each individual library.

### Alignment, Quantification and Quality Control Using MAGIC RNA-Seq Software

Eight transcriptomic datasets were analyzed in the present series of experiments. These included an SCG dataset (*n* = 4), a nodose ganglia dataset (*n* = 4), two lumbar DRG datasets (DRG batch 1, *n* = 8, and DRG batch 2, *n* = 4), a TG dataset (*n* = 3), a sciatic nerve dataset (*n* = 4), an IML dataset (*n* = 8), and a dorsal horn dataset (*n* = 2). The two batches of DRG samples were pooled and averaged. DRG batch 1, sciatic nerve (BioProject PRJNA313202) (Sapio et al., 2016), TG (LaPaglia et al., 2018), and rat muscle [gastrocnemius, PRJNA401293; quadriceps, PRJEB22693 (Sollner et al., 2017)] datasets were mined from previously published datasets (Sapio et al., 2016; LaPaglia et al., 2018). The SCG, Nodose, DRG batch 1, IML, and dorsal horn datasets were generated specifically for the present manuscript, and will be deposited in the SRA database (accessible under BioProject# PRJNA681229). Rat RNA-Seq datasets were aligned and quantified using the MAGIC pipeline (Zhang et al., 2015) and a rat genome target built using a recent RefSeq annotation (Rn6) as described previously (LaPaglia et al., 2018). This genomic target is publicly available on the MAGIC ftp server, and upon request. Calculations of read coverage, alignment percentages, and other quality control metrics are generated after sequence alignment. Quantification and normalization of gene counts was performed by MAGIC and is reported in significant fragments per kilobase of transcript per million mapped reads (sFPKM). These quantitative units are displayed in tabular form for all genes in Supplementary Table 3. Normalization of gene counts was performed as described (Zhang et al., 2015; LaPaglia et al., 2018) and includes several refinements to absorb technical variations and batch effects. Differential expression analysis was performed in MAGIC, and complete lists of significantly differential genes between each sample are reported in the Supplementary Tables. We report a score for each gene calculated from the separation of the distributions between two sample groups, and select significance of this by maintaining a false discovery rate below 5% (LaPaglia et al., 2018; Raithel et al., 2018). The differential scores for all genes measured as significant are reported in Supplementary Table 4.

### Visualizations of Ganglionic RNA-Seq Datasets

**FIGURE 1:**
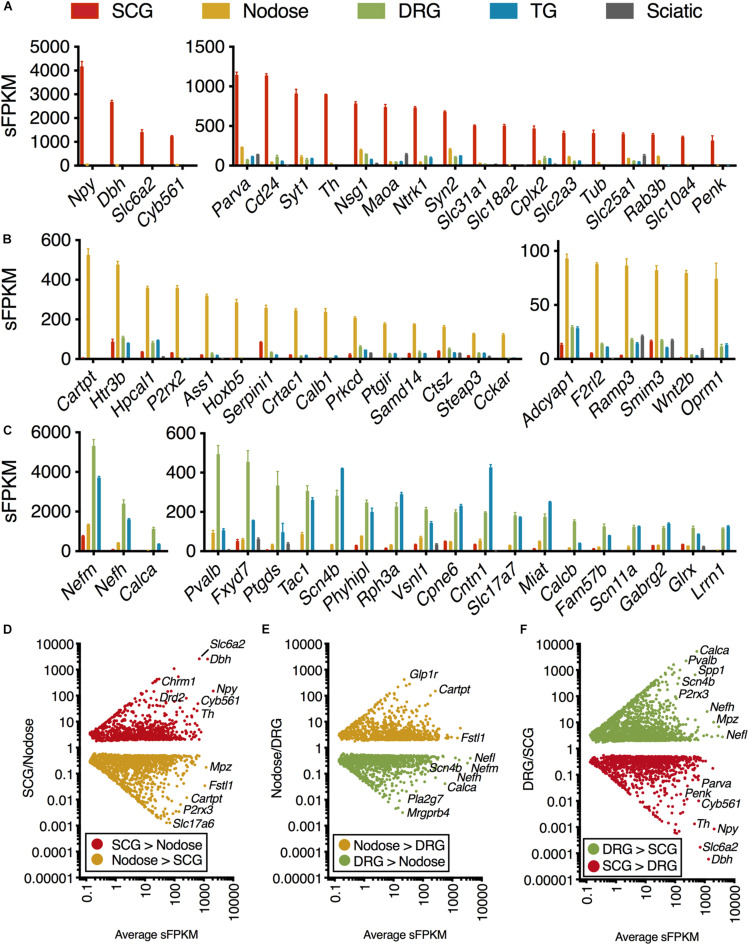
Transcriptomic signature of three peripheral ganglia. Five datasets were analyzed comprising the transcriptomes of SCG, Nodose, DRG, TG, and sciatic nerve. **(A–C)** Differential transcriptional fingerprint of the three ganglia. Genes for each of the three ganglia were filtered for ≥3 enrichment against the other 4 samples in the dataset. DRG was not filtered for enrichment relative to TG due to similarity between these sensory ganglia. SCG, *n* = 4; Nodose, *n* = 4; DRG, *n* = 12; TG, *n* = 3; Sciatic, *n* = 4. Error bars represent the standard error of the mean. **(D–F)** Overall pattern of differential genes are shown for each of the three comparisons between the three major ganglia in the present study (SCG, nodose, DRG).

Genes were ordered by sFPKM, which estimates abundance, and genes with ≥3 fold enrichment in one ganglion relative to the others were selected (Figures 1A–C). Expression ratio was calculated with the addition of a small number (0.1) added to both the numerator and denominator to reduce the impact of dividing by very small numbers. Subsequently, ER was plotted against sFPKM to select for highly expressed highly differential genes between ganglion (Figures 1D–F). The genes in Figures 1D–F are significant, with total number of signficant genes for each comparison shown in Supplementary Figure 1.

In examinations of nicotinic acetylcholine receptor subuints, possible configurations were compiled based on previous studies (Mao et al., 2006; Millar and Gotti, 2009). In the analysis of muscle, rat gastrocnemius (PRJNA401293) was selected as representative, and was re-aligned using the same aligner and genome build as other datasets in this study. Note the white break in the heatmap indicating that these data come from an outside source with different library preparation and sequencing methodology. Also note that in general subunits of the nicotonic receptors were not as strongly detected in muscle.

### Determination of Highly Selective Ganglionic Genes

Screening parameters were set to filter for highly enriched ganglionic genes. This filtration criteria was set by selecting for the highest expression ratios between each ganglion and each of the others, as well as sciatic nerve. Subsequently, we also screened for genes that were lowest expressed in any of the 32 tissues in the Human Protein Atlas. Note that the DRG and trigeminal were considered together due to their similarity in transcriptomic signature. At the bottom of the heatmap, two genes that were enriched in all three sensory ganglia (*Scn10a* and *Kcnk18*), but were not found in other tissues are shown.

### Spinal Cord Microdissections

Spinal cords were collected from male Sprague-Dawley rats (Envigo) via hydraulic ejection (Sapio et al., 2020) and the thoracic segment was sectioned into 700 μm slices using a McIlwain Tissue Chopper (Cavey Laboratory Engineering Co., Surrey, United Kingdom). Slices were laid on a glass slide and flash frozen on a steel anvil that had been precooled to −80°C. Slides containing the frozen slices were placed under a dissecting microscope on a petri dish inverted over dry ice and the IML was punched bilaterally using a flat-end 21-gauge needle. The tissue was expelled with 40 μl Qiazol (Qiagen) using a 3 mL syringe with Luer lock into a Lysing Matrix D tube (MP Biomedicals, Santa Ana, CA, United States) that was on dry ice. Samples were stored at −80°C until processing. For anatomical evaluation of the punch-based sampling, punched slices were thawed in 4% PFA and allowed to fix for 2 h. They were then incubated with Multiple Stain Solution (Poly Sciences, Inc, Warrington, PA, United States) for 30 s before being washed for ∼30 s with 70% ethanol. Each slice was imaged using an Olympus BX60 microscope. Subsequently, images were converted to diagrammatic representations of punch area by selecting a circular region corresponding to the punched area for each stained and punched section. The location of each punch was marked as a red circle on an illustration of the corresponding spinal segment using GNU Image Manipulation Program. A z-projection was then generated using ImageJ to represent the density of punched area within the diagrammatic representation of the spinal cord. The resultant image (Figure 7D) demonstrates the median dissection location in red, and the average dissection location in gray, which appears as a diffuse representation in areas where few sections were captured.

### Comparison of Peripheral and Central Myelin-Encoding Genes

A selection of myelin sheath proteins, and proteins related to myelin synthesis and function were surveyed in sciatic nerve and IML punches. Sciatic nerve samples come from a previous examination of peripheral ganglion and nerve (Sapio et al., 2016). While this selection is not exhaustive (Siems et al., 2020), we survey highly specific, and highly expressed myelin proteins such as myelin protein zero, which is a major component of the sciatic nerve transcriptome (Sapio et al., 2016). In Figure 8, expression level in sFPKM was compared between datasets, and log expression is represented as a bar to the right. Expression ratio (ER) between the two datasets is represented as a color scale (purple indicating enrichment in IML, yellow indicating enrichment in sciatic nerve). Entries are sorted by expression ratio, with the IML enriched genes at the top and the peripheral myelin genes at the bottom.

## Results

The present investigation examines the complete transcriptome of four peripheral ganglia, the IML, and the dorsal horn of the spinal cord. In the ganglionic analyses, peripheral nerve was included as a control and comparator to identify the gene signatures contributed by non-neural cells, in particular Schwann cells (Sapio et al., 2016). Enriched genes for each ganglion were examined by filtering for ≥3 fold enrichment against the other 4 samples in the dataset (Figures 1A–C). DRG was not filtered for enrichment relative to TG due to overall similarity between these sensory ganglia (Figure 1C) (Goswami et al., 2014a; Sapio et al., 2016; LaPaglia et al., 2018). As expected, the genes related to catecholamine synthesis and storage are highly expressed and highly differential in the SCG relative to other ganglia (*Dbh*, *Th*, *Maoa*, *Slc6a2*, *Slc18a2*, and *Cyb561* are all related to these functions). Some of the more prominent SCG signaling molecules are the neuropeptides Neuropeptide Y (*Npy*) and Proenkephalin (*Penk*). The nerve growth factor receptor (*Ntrk1*) is also highly expressed and differential. We also observed enrichment of other ganglion-specific specialized molecules such as the actin binding protein Parvin A (*Parva*) and the vesicular membrane protein synaptotagmin 1 (*Syt1*). In the NDG, some prominently enriched genes are the neuropeptides including Cocaine and amphetamine regulated transcript (CART) peptide (*Cartpt*) and Pituitary adenylate cyclase-activating polypeptide (*Adcyap1*). The nodose also showed enrichment of receptors such as the ATP receptor P2X purinoceptor 2 (*P2rx2*), the mu-opioid receptor (*Oprm1*), the ionotropic serotonin receptor 3B (*Htr3b*) and the protease-activated receptor 3 (*F2rl2*). In the DRG and trigeminal, there was a higher degree of similarity as these ganglia serve similar functions. These ganglia showed enrichment of neuropeptides such as the Substance P precursor preprotachykinin 1 (*Tac1*) and precursor protein of the neuropeptide calcitonin gene-related peptide beta (*Calcb*). The voltage-gated sodium channel subunits beta 4 (*Scn4b*) and alpha 11 (*Scn11a*) were also enriched in these ganglia. All three neurofilaments (light, medium and heavy chains) were also enriched in the DRG and trigeminal relative to the other ganglia (explored in more detail in Supplementary Figure 2). Notably, as described previously, there are some prominent gene differences between dorsal root and trigeminal ganglion that have been examined using RNA-Seq (Manteniotis et al., 2013; Flegel et al., 2015; LaPaglia et al., 2018). Our study confirms some of these major differences, such as the enrichment of *Trpa1* and *Trpm8* in trigeminal ganglion compared to DRG. Conversely, the precursors for CGRP (*Calca* and *Calcb*) were more abundant in DRG than trigeminal (LaPaglia et al., 2018).

The representation of all differential genes between each of the three types of ganglia in the core comparison in the present manuscript (SCG, Nodose, and DRG) are shown as scatter plots where the significant genes are plotted by fold change vs. expression level (Figures 1D–F). Note that the full table of supplementary genes is also available in Supplementary Table 4. Some highly differential, highly expressed genes are given labels in the scatter plots, such as the Calcitonin related polypeptide alpha (*Calca*) gene (which encodes the precursor protein of the neuropeptide calcitonin gene-related peptide alpha, CGRPα) in the DRG vs. SCG plot. The data in Figure 1 shows the defining differential transcriptional fingerprint for genes in each ganglion. Summaries of the number of significant genes in each comparison between these three ganglia, as well as TG and sciatic nerve are shown in Supplementary Figure 2. The sciatic nerve was examined because it contains very high numbers of myelinating Schwann cells which are, to varying extents, present within each of the peripheral ganglia examined (Sapio et al., 2016). Notably, the SCG contains the least amount of Schwann cells and myelinated fibers, and thus the lowest levels of expression of myelin genes. The inclusion of the sciatic nerve dataset allows for filtration of receptors and other gene differences attributable to Schwann cells within each ganglion which comprise a major component of peripheral nerve (Sapio et al., 2016).

**FIGURE 2:**
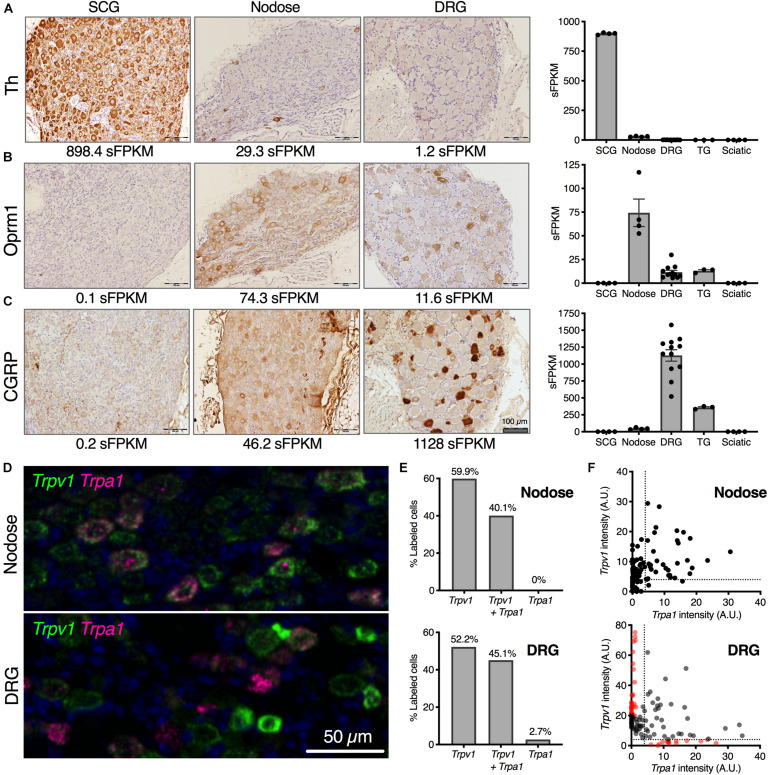
Cellular localization of selected neuronal markers from transcriptomic enrichment analysis. Anatomical correlates of transcript expression were investigated by staining for highly expressed genes of interest in SCG, nodose and DRG. **(A)** Tyrosine hydroxylase (Th) protein was examined, showing near-ubiquitous expression among SCG neurons (898.4 sFPKM), with no expression in nodose or DRG. **(B)** Oprm1 was expressed very strongly in nodose, with detectable protein level expressed throughout the ganglion (74.3 sFPKM) with fewer cells per section expressing Oprm1 in the DRG (11.6 sFPKM). **(C)** CGRP was largely specific to the DRG (1128 sFPKM of the precursor, *Calca*) with substantially lower levels in nodose (46.2 sFPKM). Trpv1 and Trpa1 are both expressed in nodose and DRG where they respond to noxious stimuli. **(D)** The expression pattern was examined in both DRG and nodose using multiplex in situ hybridization. A range of expression levels and colocalization was noted and these parameters are analyzed in panels **(D,E)**. **(E)** The expression pattern of *Trpa1* was highly overlapping with *Trpv1*, with about between 40 and 60% of the cells in either ganglion expressing *Trpv1* alone, or *Trpv1* with *Trpa1*. While there was a small population of *Trpa1*+ cells without *Trpv1* in rat DRG, this was not observed in the nodose. **(F)** Quantitatively, the expression of the two TRP channels was more divergent in the DRG as well, showing populations of cells with high levels of one or the other channel (red dots).

One highly expressed marker gene from the transcriptomic signature of each ganglion was examined using immunohistochemistry (Figures 2A–C). Tyrosine hydroxylase (*Th*), the first enzyme in catecholamine biosynthesis is highly expressed in the SCG (894.4 sFPKM) and nearly all neurons in this ganglion are TH-positive. In contrast, TH shows sparse staining in the NDG, which is also reflected as lower overall expression (29.3 sFPKM) (Figure 2A). In the rat DRG, *Th* is comparatively low (1.2 sFPKM), and as a consequence, no staining is observed. However, other studies have described infrequent sporadic Th+ staining in rat DRG consistent with the low transcript abundance (Price and Mudge, 1983). Note that this is distinct from mouse which contains high amounts of Th (Brumovsky et al., 2006, 2012; Brumovsky, 2016), an observation which has also been confirmed using RNA-Seq studies (Goswami et al., 2014a; Usoskin et al., 2015). As is noted in several of these studies, the functional significance of tyrosine hydroxylase in DRG is still being investigated, with several theories proposed for the role of this enzyme. Tyrosine hydroxylase is often part of a biosynthetic pathway with other enzymes, the presence of which may also be interesting to investigate further in future studies. In the current study, we further examined several enzymes in the catecholamine biosynthetic and degradative pathways, and did not observe robust evidence for expression of additional biosynthetic steps past DOPA in pooled L4, L5, L6 DRGs. Dopamine beta hydroxylase (*Dbh*) and L-amino acid decarboxylase (*Ddc*) were absent from the rat lower lumbar DRGs (Supplementary Figure 8) whereas expression of the degradative enzymes was variable among the ganglia. Interestingly, among the monoamine oxidase enzymes *Maoa* was highly expressed in the SCG whereas *Maob* was more robustly expressed in the sciatic nerve most likely in the Schwann cells (Sapio et al., 2016). The mu-opioid receptor (*Oprm1*) was selected as one gene in the transcriptomic signature of the nodose. The mu-opioid receptor is expressed in many neurons of the nodose ganglia (74.3 sFPKM), but expressed in only a subset of DRG neurons (11.6 sFPKM; Figure 2B), and was absent from the SCG. This highlights the more ubiquitous expression in the nodose relative to the DRG. Calcitonin gene-related peptide (CGRP) is highly expressed in peptidergic neurons of the DRG, showing dense somatic staining in multiple neurons, which is reflected by the very high expression of the gene encoding the peptide precursor, *Calca* (1,128 sFPKM; Figure 2C). In the nodose, some CGRP staining is observed, but the immunoreactivity is less dense, and this is reflected in the much lower expression of the encoding gene (46.2 sFPKM).

We noted the enrichment of several nociceptive receptors and ion channels in the NDG, and further examined the distribution of two such channels in detail. Two nociceptive transducing ion channel receptors, Transient receptor potential cation channel subfamily V member 1 (*Trpv1*) and subfamily A, member 1 (*Trpa1*), were analyzed in DRG and NDG using *in situ* hybridization (Figures 2D,E). TRPV1 is a major thermosensing ion channel in the sensory ganglia, while TRPA1 is a major chemosensory ion channel. TRPV1 is also a marker of nociceptive neurons in the DRG and activation of this channel has been used to isolate these neurons for transcriptomics (Goswami et al., 2014a; Isensee et al., 2014). In both ganglia, these sensory transducer ion channels have a high degree of correlation in the level of expression, although we observed more overlap between *Trpv1* and *Trpa1* in the NDG, and more distinct labeling in the DRG. For example, *Trpa1* in the nodose was always observed with *Trpv1* (Figures 2D–F). Cells with predominant expression of either *Trpv1* or *Trpa1* by *in situ* hybridization are indicated in red on the scatter plots in Figure 2E, and were only seen in the DRG.

**FIGURE 3:**
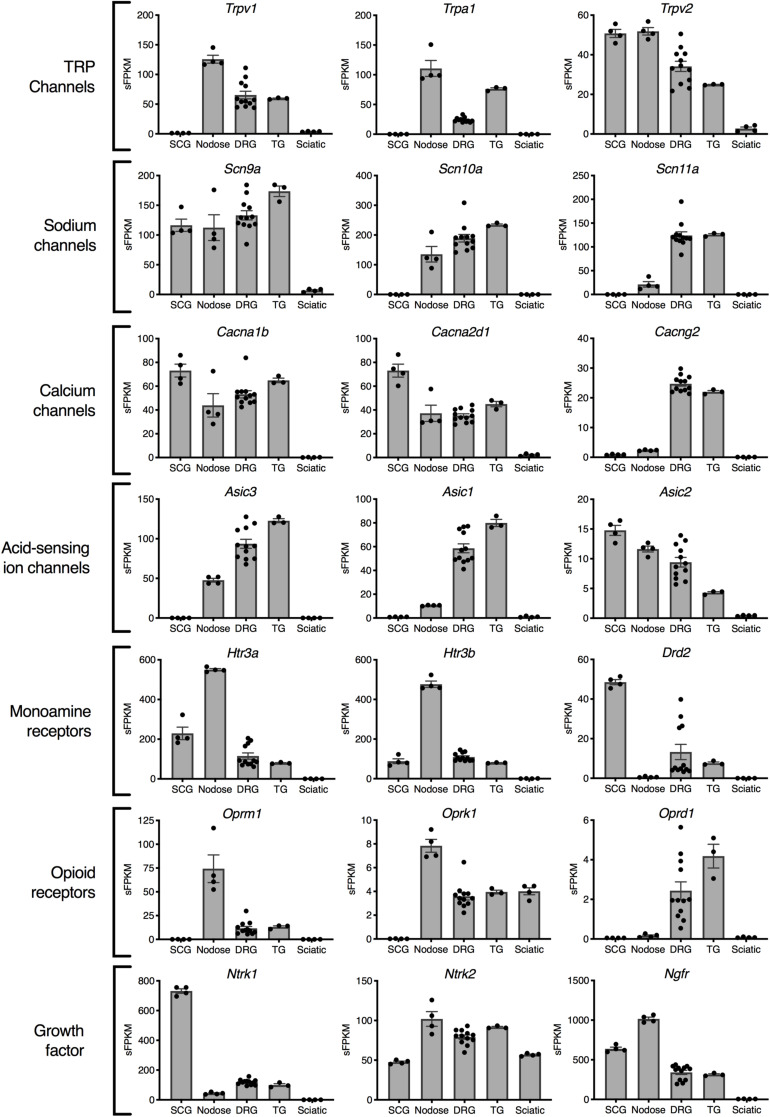
Analgesia targets and nociceptive genes. Selected genes are highlighted representing examples from families of genes relevant to nociception and analgesia. In general, the nodose ganglion expresses very high levels of most nociceptive molecules including the TRP channels: *Trpv1* and *Trpa1*, the opioid receptors *Oprm1* and *Oprk1*, and the serotonin receptors *Htr3a* and *Htr3b*. Of the voltage-gated sodium channels currently being studied for their selective expression in nociceptive neurons, Na_V_1.7 (*Scn9a*) and Na_V_1.8 (*Scn10a*) included the nodose, while Na_V_1.9 (*Scn11a*) was selective for DRG and trigeminal.

Gene expression profiles across these peripheral ganglia were examined to focus on expression of analgesic drug targets. In this analysis, drugs that act peripherally on nociceptive primary afferents were prioritized for examination. This analysis examines the presence of these drug targets on each of the ganglia, and could be used to predict side effect profiles. The primary actions of many of these drugs is on the DRG and/or trigeminal ganglion, which are responsible for the transduction of noxious stimuli into pain responses. In general, many analgesic drug target genes are highly expressed in all three sensory ganglia (DRG, trigeminal and nodose). Notably, several of the genes examined in Figure 3 were enriched in the nodose relative to DRG and/or trigeminal. The first four rows show categories of ion channel genes. Transient receptor potential ion channels were identified in nodose and DRG, with the Transient receptor potential vanilloid receptor 2 showing strong expression in SCG (comparable to sensory ganglia). Among voltage-gated sodium ion channel subunits, *Scn9a* (which encodes Na_V_1.7) was found in all four ganglia, *Scn10a* (Na_V_1.8) was found in roughly equal amounts in DRG, trigeminal and nodose, and *Scn11a* (Na_V_1.9) was enriched in DRG and TG. This indicates that *Scn11a* is likely the most specific to DRG and TG, and that Na_V_1.8 and Na_V_1.9 might offer the most analgesic specificity. Of note, *Scn8a* (Na_V_1.6) is also highly enriched in DRG and trigeminal, where it has been implicated in cold allodynia (Deuis et al., 2013). Some calcium channel genes such as the Voltage-dependent calcium channel subunit α2δ1 (*Cacna2d1*) were expressed more strongly in the SCG (Figure 3). Note the high expression and enrichment of the nerve growth factor receptor, TrkA (*Ntrk1*) in the SCG relative to sensory ganglia. Several broad families of potential target genes are additionally explored using heatmaps, including a much broader selection of genes encoding peptide precursors, G-protein coupled receptors, catalytic receptors, and ligand-gated and voltage-gated ion channels (Supplementary Figures 3–7).

**FIGURE 4:**
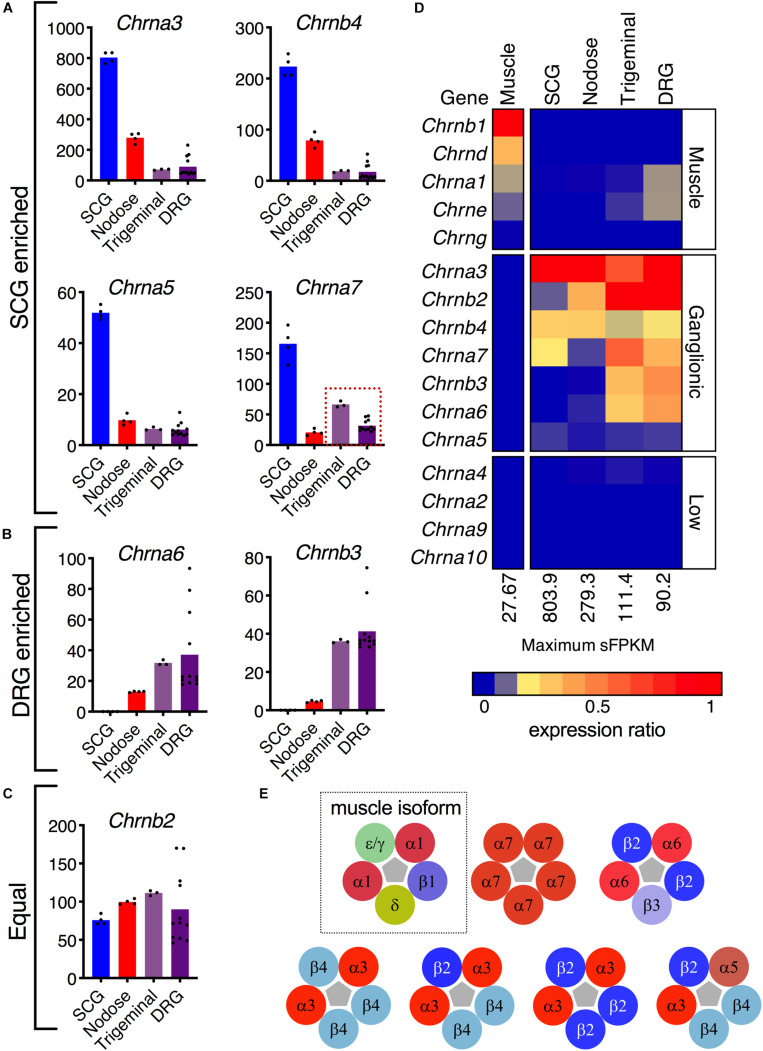
Nicotinic receptor composition. **(A)** The α3, α5, α7, and β4 subunits are enriched in superior cervical ganglion. **(B)** The sensory ganglia demonstrate enrichment of α6 and β3 subunits. **(C)** The β2 subunit is robustly expressed in the four ganglia. **(D)** Expression of each gene was normalized to the highest sFPKM in each tissue, with blue corresponding to no expression, and red to highest level of expression. The flame scale at the bottom of the heatmap indicates expression ratio (from 0.0 to 1.0). **(E)** Pentameric aggregates forming functional receptors. The α1β1δε/γ pentamer is the functional ligand-gated channel found in the neuromuscular junction (dotted outline). The α7 homomer can be found in modulatory interneurons within the dorsal horn, as well as throughout the brain. The α6β2β3 pentamer is found exclusively in sensory ganglia. The α3β4 pentamer is found throughout the nervous system and is the most common nAChR in the superior cervical ganglion. The α3 subunit can also form pentamers with β2 and β4. Though rare, a functional α3α5β2β4 receptor in the SCG has also been described.

Nicotinic receptors are explored in depth because of recurrent efforts to develop specific analgesic and antitussive drugs targeted at subunits of these receptors (Figure 4) (Dicpinigaitis et al., 2014; Naser and Kuner, 2018; Tao et al., 2019). The topic of cholinergic receptors in the DRG was also examined in our previous study of specific sorted cell populations of DRG neurons in the mouse. In that study, *Chrna6* was enriched in the nociceptive *Trpv1* lineage of DRG neurons, while *Chrna7* was much higher in *Trpv1* non-lineage cells (Goswami et al., 2014a). These previous findings prompted an examination of cholinergic receptors in rat peripheral ganglia. In this analysis we expand the search to all subunits of the nicotinic acetylcholine receptor to further explore the specificity of these subunits to DRG relative to other ganglia and muscle. Several subunits of nicotinic acetylcholine receptors are enriched in SCG (*Chrna3*, *Chrnb4*, *Chrna5*, and *Chrna7*) (Figure 4A), while *Chrna6* and *Chrnb3* are enriched in DRG (Figure 4B). Examination of the pentameric active nicotinic receptors revealed some possible candidate configurations formed in these tissue types based on comparison to known configurations and tissue distributions of nicotinic receptors (Dussor et al., 2003; Millar and Gotti, 2009). Nicotinic receptors at the neuromuscular junction contain different subunits from those in nervous tissue. A previously published dataset of muscle was examined to confirm detection of the five muscle subunits in this tissue (Figure 4D). Using heatmaps, we delineate the possible pentameric forms of nAChRs in these tissues (Figure 4D). In aggregate, this describes the full transcriptomic repertoire of nicotinic receptors in these tissues, based on known configurations of subunits (Figure 4E). Note that the first configuration containing epsilon (*Chrne*) or gamma (*Chrng*) (green) and delta subunits (gold) is the configuration found at the neuromuscular junction.

**FIGURE 5:**
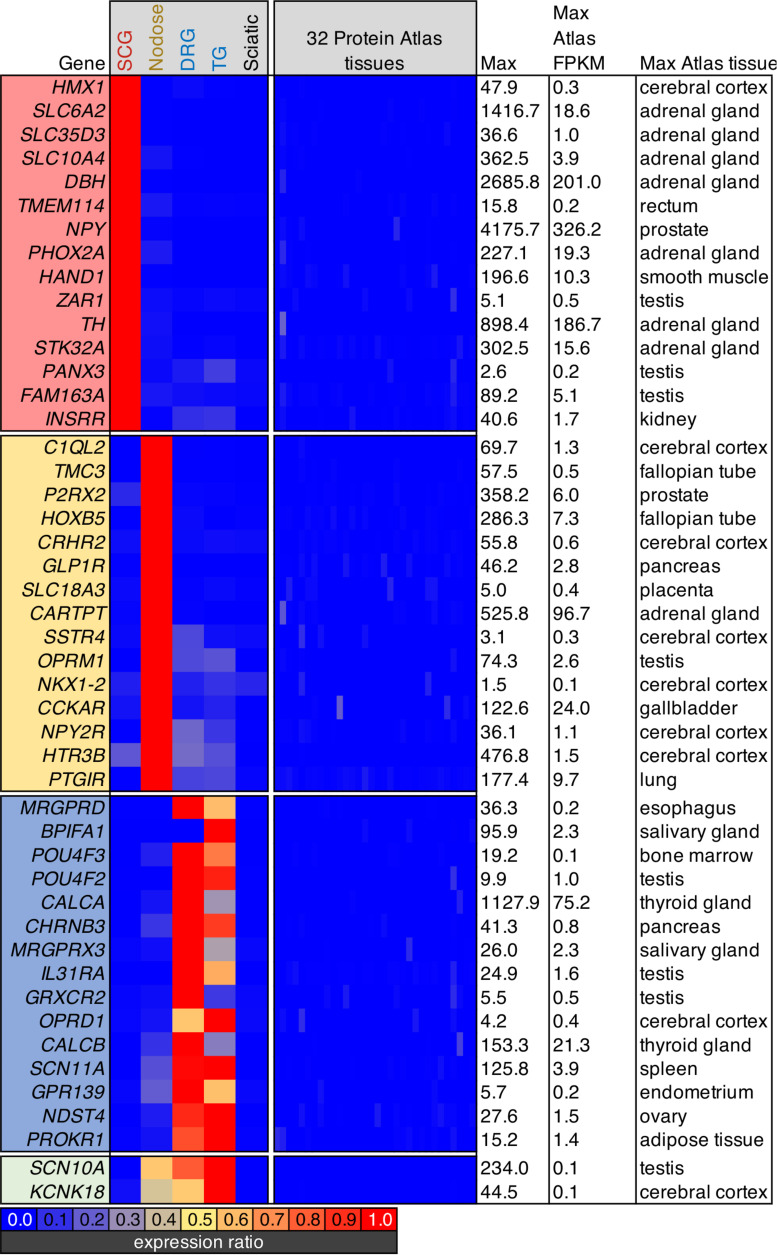
Analysis of body-wide enrichment of ganglia-specific genes. Highly enriched genes were examined for each of the three major ganglion in the present study by comparing the levels of expression to the human protein atlas dataset as described previously (Burbelo et al., 2016). Signaling molecules in the SCG such as tyrosine hydroxylase (*Th*) and neuropeptide Y (*Npy*) were prominent. Within the nodose ganglion, several markers of satiety-related functions were identified, including the cholecystokinin receptor, *Cckar*, and the somatostatin receptor *Sstr4*, as well as the receptor for glucagon-like peptide (*Glp1r*). The DRG showed an unexpected signature because of the high degree of overlap in nociceptive markers between DRG and nodose. Notably, while other opoid receptors are prominent in nodose, the delta opioid receptor (*Oprd1*) was specific for DRG.

In order to examine specific gene signatures of peripheral ganglia, we compared gene expression in each ganglia to RNA-Seq data from 32 tissues in the Human Protein Atlas, selecting for the most highly enriched genes in SCG, nodose, or DRG relative to all other body tissues examined (Figure 5). Again, this provides a transcriptomic perspective for drug development and a capacity to evaluate potential on- and off-target side effects for the purpose of drug development. For each ganglion, we examine a selection of highly enriched genes which show little expression in the Human Protein Atlas. In the SCG, the highly enriched genes include synthetic enzymes for biogenic amine transmitters such as tyrosine hydroxylase (*Th*) and dopamine beta hydroxylase (*Dbh*), and plasma membrane and vesicular catecholamine reuptake. Also identified in the SCG was the H6 homeobox 1 transcription factor (*Hmx1*), which has been identified as a regulator of TrkA expression and noradrenergic fate in sympathetic neurons (Furlan et al., 2013). Within the nodose, several of the genes identified in this analysis were peptide receptors such as the mu-opioid receptor (*Oprm1*), somatostatin receptor 4 (*Sstr4*), and the cholecystokinin A receptor (*Cckar*). Notably, several of the genes enriched in the nodose have an apparent involvement in the regulation of satiety, including genes coding for the glucagon-like peptide receptor (*Glp1r*), a neuropeptide Y and PYY receptor (*Npy2r*) and the CCK-A receptor (*Cckar*) (see discussion). Another notable receptor was the prostacyclin/PGI2 receptor (*Ptgir*), which was strongly detected in NDG (177.4 sFPKM). This is notable given that PGEI2 signaling is an established regulator of baroreceptor function (Zucker et al., 1988). Also notable in the NDG was the enrichment of the Homeobox B5 (*Hoxb5*) transcription factor, which has previously been reported to be enriched in nodose neurons relative to jugular ganglion neurons (Wang et al., 2017). In the DRG, some of the most enriched genes were neuropeptide precursor genes such as Preprotachykinin-1 (*Tac1*) and *Calca* which are precursors for substance P and CGRP, respectively. While the mu-opioid receptor is expressed in both nodose and DRG (Figures 2, 3), *Oprm1* shows enrichment in the nodose, while *Oprd1* shows enrichment in the DRG. This is also reflective of the very low levels of these receptors in most other tissues, which causes a strong degree of ganglionic enrichment. Finally, *Scn10a* and *Kcnk18* were highlighted (green box, bottom of Figure 5). These two ion channels are expressed in nodose, DRG and TG, with highest expression seen in the trigeminal ganglion, and are highly enriched compared to other tissues (LaPaglia et al., 2018).

**FIGURE 6:**
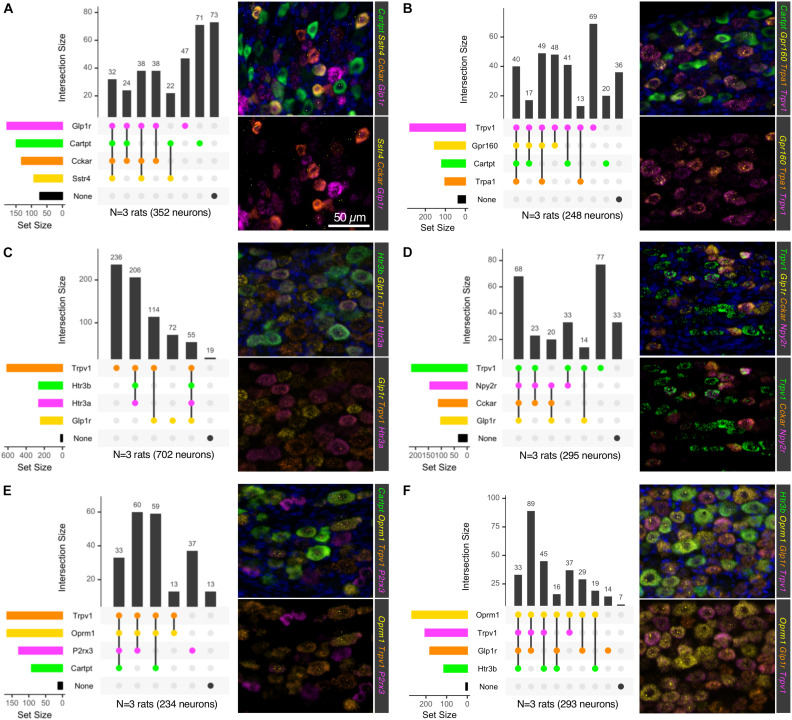
Multiplex *in situ* hybridization of nodose ganglion to identify vagal afferent populations. Six 4-plex *in situ* hybridizations were performed on nodose ganglion sections **(A–F)**. Staining positivity was determined based on a thresholded subtracted image, and cells were counted for all four labels. Intersections of staining positivity were plotted in a matrix indicating number of cells in each grouping. Marker genes were chosen based on known functions, such as *Glp1r*, which is known to induce nausea, and suppression of food intake. Similarly, *Cckar* has also been studied as a satiety-promoting receptor. Other receptors such as the PYY receptor *Npy2r* and the somatostatin receptor 4 (*Sstr4*) were also examined because of their likely role in satiety in these afferents. Conversely, other receptors involved in sensing of noxious stimuli such as *Trpv1, P2rx3*, and *Trpa1* were also examined. **(A)** The neuropeptide precursor transcript Cartpt was examined alongside several largely satiety-related or **(B)** nociceptive transducing molecules. **(C)** Ionotropic serotonin receptors (*Htr3a* and *Htr3b*) were examined alongside *Glp1r* and *Trpv1*. **(D)** *Trpv1* was examined with the putative satiety-related transcripts *Glp1r, Cckar* and *Npy2r*. **(E)** *Trpv1*, the mu-opioid receptor (*Oprm1*) and the purine receptor transcript *P2rx3*, which are each important for nociceptive processes were analyzed alongside *Cartpt*. **(F)** *Trpv1* and *Oprm1* were analyzed in relation to *Htr3b* and *Glp1r*. In each of these stains, markers of nociceptive transducing properties and those known to signal in other functions were evaluated to ascertain the degree of overlap between these two broad functions in the nodose ganglion. For each 4-plex stain, a representative field is shown, with a 3-plex version (lower panels) for clearer visualization. Scale bar (50 mm) applies to all images in the figure.

Using six 4-plex fluorescent *in situ* hybridization probe sets, we examined the cell types of the NDG (Figure 6) for combinatorial panels of these highly enriched genes. Intersections of positivity for each of the 4 markers were plotted in a matrix format, showing number of cells belonging to a category represented by the bars above the matrix, and number of cells positive for a single label represented by the colored bars to the left of the matrix. For each 4-plex stain, a representative field is shown for 4-plex labeling (Figure 6). Because 4-plex is difficult to see in the overlay, the same image is shown below it as a 3-plex stain, subtracting a bright and/or overlapping signal for visualization. Marker genes were selected based on predicted functions of individual cell types. These included signaling receptors implicated in satiety and/or nausea such as Glucagon-like peptide-1 receptor (*Glp1r)*, *Cckar* and *Sstr4*. In particular, the somatostatin receptor Sstr4 has been proposed as an analgesic target, and as a regulator of pain and inflammation, prompting an investigation of its cellular expression (Pan et al., 2008; Van Op den Bosch et al., 2009; Schuelert et al., 2015). Additionally, genes involved in interoception such as *Trpv1*, *Trpa1*, and P2X purinoceptor 3 (*P2rx3*) were examined. Genes were also selected in some cases based on high level expression or relevance to the biomedical field, such as the two ligand-gated ionotropic serotonin receptors 5HT-3A and 5HT-3B (*Htr3a* and *Htr3b*) and the mu-opioid receptor (*Oprm1*). One goal of this series of staining experiments was to assess the co-expression of nociceptive and satiety-related genes. Neurons expressing *Glp1r* frequently expressed receptors such as *Cckar* and *Sstr4*, as well as the neuropeptide precursor gene *Cartpt* (Figure 6A), although we also observed a subset of cells expressing *Glp1r* or *Cartpt* alone. Notably, using these four probes, a large percentage of neurons were unlabeled. When examining *Trpv1*, *Trpa1* and *Cartpt* together, we observed cells expressing *Cartpt* alone, *Trpv1* alone, and triply labeled cells that also contained the gene encoding the recently de-orphaned G-protein coupled receptor *Gpr160* (Figure 6B). Gpr160 was examined because it is enriched in sensory ganglia, where it has been proposed to play a role in neuropathic pain (Yosten et al., 2020). In order to investigate the coincidence of nociceptive genes such as *Trpv1* and satiety genes such as *Glp1r*, these two genes were stained together with the ionotropic serotonin receptors *Htr3a* and *Htr3b*. Either *Trpv1* or *Glp1r* labels virtually all neurons in the NDG, with a small percentage (∼8%) of quad-positive neurons seen for this labeling scheme (Figure 6C). Notably, Trpv1 labeled the majority of neurons, with the most prevalent subpopulations being *Trpv1* alone, *Trpv1* + *Htr3a* + *Htr3b*, and *Trpv1* + *Glp1r* (Figure 6C). This shows the potential for functional overlap of nociceptive neurons containing *Trpv1* and satiety/nausea sensing neurons expressing *Glp1r*. This was further explored by staining with additional satiety genes such as *Npy2r* and *Cckar* (as discussed above). In this experiment, *Trpv1*/*Glp1r* co-positive neurons frequently expressed *Npy2r* and/or *Cckar* (Figure 6D). Finally, the mu-opioid receptor was also examined, with the majority of *Oprm1*+ cells also expressing *Trpv1* (Figures 6E,F). These cells also frequently expressed the purine receptor *P2rx3*, although there was also a subclass of P2rx3 neurons without *Oprm1* or *Trpv1*. Notably, *Trpv1* and *Oprm1* were both very prevalent, staining the majority of neurons in the ganglion. Precise numbers of cells and sections counted are shown in Supplementary Table 2.

**FIGURE 7:**
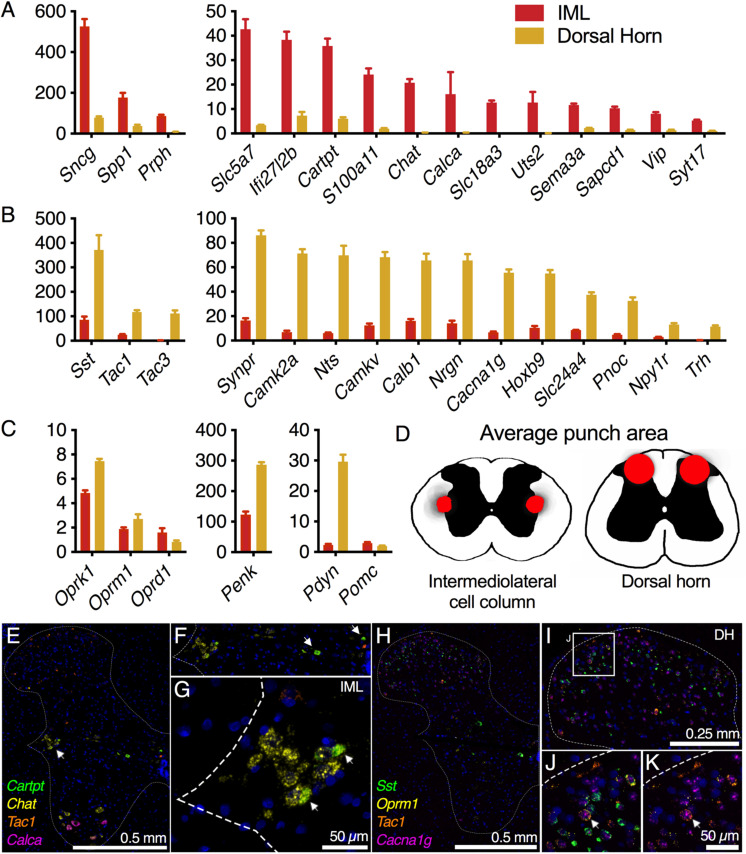
Intermediolateral cell column (IML) and dorsal horn (DH) spinal cord microdissection. Micropunches of the IML (*n* = 8) or DH (*n* = 7) were collected to examine specific gene expression in these two subregions of the thoracic (IML) and lumbar (DH) spinal cord in the rat. Each of these regions is defined by a small number of specific genes reflecting the specific signaling pathways employed by these regions. **(A)** In the IML, cholinergic signaling components such as choline *O*-acetyltransferase (*Chat*) and choline transporters (*Slc5a7*, *Slc18a3*) are prominently enriched, reflecting the specific utilization of cholinergic signaling by neurons in this cell column. The IML also specifically produces other signaling molecules such as the CGRP and urotensin precursor genes (*Calca* and *Uts2*). **(B)** In the dorsal spinal cord, several specific transmitter molecules such as somatostatin (*Sst*), tachykinin 1 and 3 (*Tac1*, *Tac3*) and pronociceptin (*Pnoc*) were observed. **(C)** To focus on opioid signaling specifically, several opoid receptors and precursor proteins were analyzed. None of the opioid receptor genes were strongly differential between these two spinal regions, while prodynorphin (*Pdyn*) was strongly enriched in the DH, consistent with its superficial laminar distribution. **(D)** Subsequent to micropunch, stained sections were analyzed for punch location, and an aggregate of all punch locations is shown for both IML and DH punches. **(E–G)** Marker genes for the IML (*Chat*, *Cartpt*) were examined alongside a marker of the dorsal horn (*Tac1*), while *Calca* was largely in ventral motor neurons. **(H–K)** Four DH markers (*Sst*, *Oprm1*, *Tac1*, and *Cacna1g*) were examined together, with all four genes showing largely separate staining patterns.

Pre- and postsynaptic connectivity at the level of the spinal cord is analyzed in Figure 7. Centrally projecting sensory axons route information from peripheral neurons in DRG to make synaptic contacts with second-order neurons in spinal cord. For the sympathetic system, the post-ganglionic sympathetic neurons in the ganglia receive innervation from the preganglionic sympathetic neurons in the IML, which are found in discontinuous clusters in the thoracic spinal cord (Rando et al., 1981). To examine the signaling between spinal and ganglionic neurons, the IML and dorsal spinal cord were subdissected using micropunch followed by RNA-Seq (Figure 7). IML-enriched genes were selected relative to dorsal horn. These included several components of the neurotransmitter apparatus such as choline acetyltransferase (*Chat*), the high-affinity choline transporter (*Slc5a7)*, and the neuropeptide precursor genes, Cocaine- and amphetamine-regulated transcript (*Cartpt*) and Vasoactive intestinal peptide (*Vip)* (Figure 7A). The CGRP precursor gene *Calca* was also identified as highly enriched in the IML relative to dorsal horn, and the CGRP peptide is thought to be made by a small number of cells in the IML region (Yamamoto et al., 1989; Dagerlind et al., 1994). In the section illustrated (Figure 7E) we did not detect a *Calca*+ IML neuron. However, staining of the whole spinal cord showed dense labeling of large *Calca+* motor neuron perikarya in ventral horn (Figures 7A,E). In the dorsal cord, several neuropeptide precursor genes were strongly enriched including Somatostatin (*Sst*), Preprotachykinin-1 (*Tac1*), Preprotachykinin-3 (*Tac3*), and Neurotensin (*Nts*), as well as Calcium/calmodulin-dependent protein kinase IIa (*Camk2a*) and the related pseudokinase gene *Camkv* (Figure 7B). A specific examination of opioid receptors and peptide precursor proteins revealed comparable amounts of all three opioid receptors in the IML and dorsal horn. Proenkephalin (*Penk*) and prodynorphin (*Pdyn*) were enriched in the dorsal spinal cord, with *Pdyn* enriched to a greater extent (Figure 7C). The accuracy of the punch was assessed by staining the sections after punching and creating a punch density diagram showing the median punch area (red) with the average punch area (gray) in the background (Figure 7D). These diagrams show the anatomical distribution of the punches, which were localized around the medial aspect of the dorsal horn, or encompassing the region surrounding the IML. Two 4-plex combinations of markers were used to localize IML-enriched and DH-enriched transcripts in specific cell populations (Figures 7E–K). Three IML-enriched marker genes (*Cartpt*, *Chat*, *Calca*) were examined in combination with a DH-enriched gene (*Tac1)* (Figures 7E–G). Note that a receptor for CART peptide (Gpr68, 14 sFPKM) is expressed in the SCG, reflecting the transmission of sympathetic information from IML to SCG. Of these genes, *Chat* was a robust marker of IML neurons, whereas *Cartpt* was co-expressed with *Chat* in a restricted subpopulation. The high level of *Chat* is consistent with the encoded enzyme’s function in generating presynaptic stores of acetylcholine. Separately, four markers of DH neurons were examined together (*Sst*, *Oprm1*, *Tac1*, *Cacn1g*) (Figures 7H–K). These populations were largely separate, although one cell (arrow, Figure 7J) is highlighted that is positive for all four markers. The low-voltage-activated T-type calcium channel α1G subunit (*Cacna1g*) is broadly expressed in the majority of cells in the dorsal horn, while *Tac1* appears largely localized to the superficial layers (Figures 7H–K). The transcript for *Oprm1* is broadly distributed throughout the spinal cord but there is a distinct set of positive neurons spread in superficial laminae I and II. The peptide precursor transcript *Sst* is primarily localized to laminae II, with a small population of these cells being quad-positive.

**FIGURE 8:**
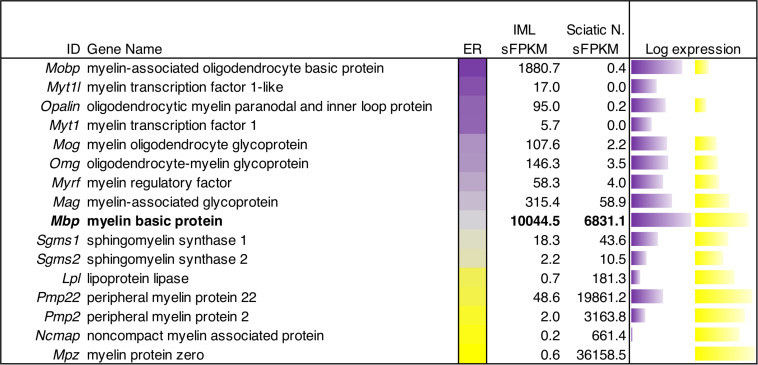
Quantitative gene expression analysis of myelin genes in peripheral and central myelinating cells. The central bar is a flame scale based on the log of the ratio, and sorted by enrichment in IML relative to sciatic nerve. The genes in this panel of myelin-encoding proteins generally show enrichment in peripheral (sciatic) nerve tissue or central (IML) nervous tissue. Myelin-associated oligodendrocyte basic protein was highly detected (∼1881 sFPKM) in IML, but barely detectable in sciatic nerve (0.4 sFPKM). Conversely, Myelin protein zero was the highest expressed (36,000 sFPKM) peripheral myelin component, and was highly enriched in sciatic nerve tissue compared to IML. Myelin basic protein (*Mbp*) is highlighted as an example of a shared myelin component between peripheral and central myelinating cells with approximately equal levels in both tissues (∼7000–10,000 sFPKM).

One distinction between peripheral and central tissues is the production of the myelin sheath by oligodendrocytes (centrally) and Schwann cells (peripherally) leading to differences in the overall protein compositions of these two distinct types of myelin. We examined the gene expression in ganglionic (peripheral) and spinal (central) tissue to compare expression of the myelin components. These signatures were overlapping to some degree, with Myelin basic protein (*Mbp*) showing very high expression in both myelin-producing tissues. By contrast, genes such as Myelin-associated oligodendrocyte basic protein (*Mobp*), Oligodendrocytic myelin paranodal and inner loop protein (*Opalin*) and Myelin oligodendrocyte glycoprotein (*Mog*) were highly enriched in central tissue. Conversely, Myelin protein zero (*Mpz*), Peripheral myelin protein 2 (*Pmp2*), and Peripheral myelin protein 22 (*Pmp22*) were enriched in peripheral myelin-producing tissues (Figure 8).

**FIGURE 9:**
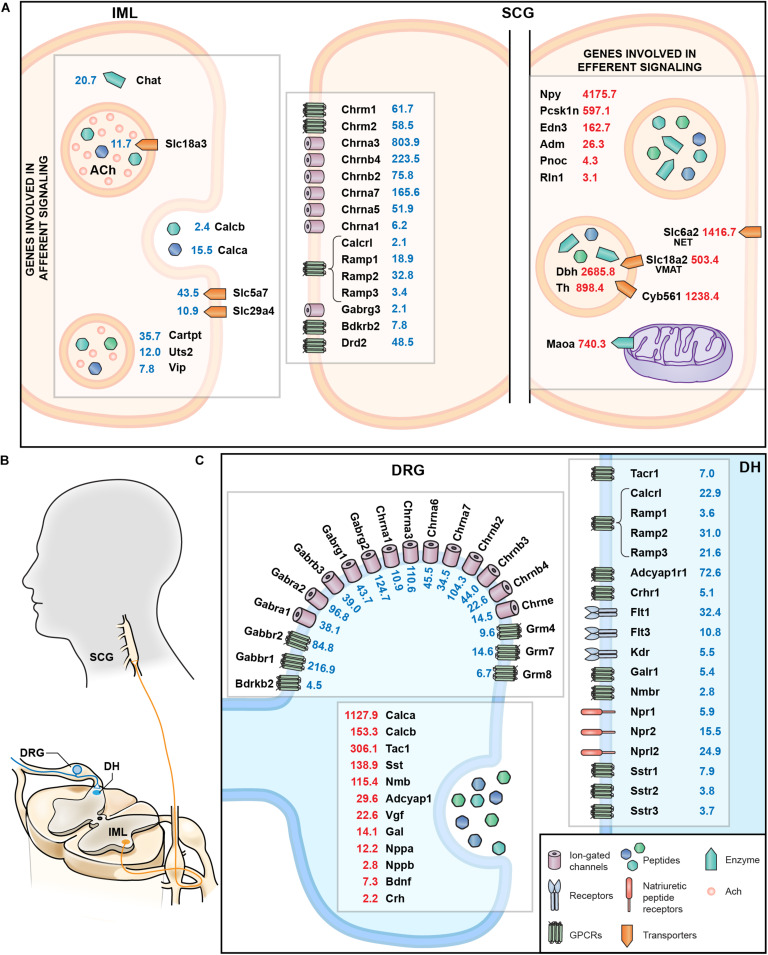
Summary of molecular interfaces. **(A)** Presynaptic signaling between IML and SCG is primarily cholinergic. Receptors for bradykinin, dopamine, and GABA at the post-synaptic SCG indicate input from structures other than the IML. Genes involved in norepinephrine metabolism are enriched in SCG, as are peptides whose receptors are found in distant structures, such as relaxin (*Rln1*). **(B)** Anatomical schematic showing connectivity between DRG and dorsal horn and IML and SCG. **(C)** The interface between DRG and dorsal horn reveals release of several neuropeptides such calcitonin gene related peptides (*Calca* and *Calcb*) as well as cognate receptors in the dorsal horn (*Calcrl*). Other examples include *Tac1* released from DRG onto *Tacr1*+ cells in the dorsal horn, and natriuretic peptide release (*Nppa*, *Nppb*), which interacts with the natriuretic peptide receptors (such as *Npr1* and *Npr2*).

A summary schematic showing genes involved in afferent and efferent signaling in the sympathetic circuit from the IML to the SCG are examined comprehensively in Figure 9A. This figure highlights both the localization as well as the quantitative expression levels from the RNA-Seq data. This includes presynaptic signaling molecules such as *Chat* and ACh transporters involved in cholinergic signaling onto the SCG. As a complement, the cholinergic receptors of the SCG were examined, as well as receptors for CGRP, GABA, bradykinin and dopamine (such as the D2 dopamine receptor, *Drd2*). The SCG performs efferent signaling to end organs by releasing peptides that include neuropeptide Y (*Npy*) and endothelin 3 (*Edn3*) as well as monoamines synthesized by dopamine beta hydroxylase (*Dbh*) and tyrosine hydroxylase (*Th*) all of which are highly expressed in the SCG. Signaling between DRG and dorsal horn (Figure 9B) was also examined, with a focus on cholinergic and GABA receptors on the DRG presynaptic endings, as well as the neurotransmitters released from DRG onto dorsal horn neurons and the postsynaptic receptors in the dorsal horn (Figure 9C).

## Discussion

This report presents comprehensive molecular fingerprints of superior cervical, nodose, dorsal root and TG both individually and in relationship to one another. We focus on examining signaling molecules and receptors involved in intercellular communication between these ganglia and the spinal cord, peripheral organs, and external stimuli. Additionally, we focus on transcriptional signatures of nociceptive afferents in the DRG relative to the other peripheral ganglia, and to transcriptomic signatures throughout the body. Further, we contrast the sensory transcriptomic signature with those of the sympathetic efferents of the SCG. These examinations reveal extensive overlapping nociceptive signatures in the DRG and trigeminal ganglion, but also in the NDG. This ganglion exhibits a remarkable enrichment of noxious stimulus transducing molecules relative to the DRG suggesting that many pharmacological manipulations directed at DRG nociceptors may also act similarly, or in a more pronounced way, upon vagal afferents.

Identifying potential drug targets with highly specific expression patterns allows for the creation of powerful therapeutic agents with minimal side effects. One example of this approach comes from the work on sodium voltage-gated channel α subunit 9 (*Scn9a*, Na_V_1.7), which is required for action potential generation in nodose (Muroi et al., 2011) and dorsal root (Schmalhofer et al., 2008) ganglion, but is also highly expressed in the superior cervical ganglia (Figure 3). In contrast, sodium voltage-gated channel α subunit 10 (*Scn10a*, Na_V_1.8) is absent from the SCG, while sodium voltage-gated channel α subunit 11 (*Scn11a*, Na_V_1.9) is present only in the DRG and trigeminal (Figure 3 and Supplementary Figure 7). The *Scn11a* transcript is also highly enriched in DRG relative to all the other tissues in the protein atlas (Figure 7), making it highly specific for the DRG. Similarly, *Scn8a* was also enriched in DRG and trigeminal. Most efforts have been aimed at developing Na_V_1.7 and 1.8 antagonists, in part driven by loss and gain of nociceptive function human mutations (Dib-Hajj et al., 2005, 2007, 2013; Goldberg et al., 2007). However, given the degree of enrichment, the level of expression, and similar human mutation observations, Na_V_1.9 blockers may provide more nociceptive selectivity than the other two DRG voltage-gated sodium channels (Dib-Hajj et al., 2002, 2015; Leipold et al., 2013; Zhang et al., 2013). Na_V_1.6 has been implicated in cold allodynia (Deuis et al., 2013), and may also be a good candidate for clinical development based on its transcriptomic profile. The voltage-gated calcium channel subunit α1B (*Cacna1b*) and auxiliary subunit α2δ1(*Cacna2d1*), which are the major targets of ziconotide and gabapentin, respectively, are also expressed in the SCG (Figure 3). This shared expression between DRG and SCG is of interest considering the effects of gabapentin on complex regional pain syndrome type I (previously termed reflex sympathetic dystrophy) a chronic neuropathic pain disorder thought to have an overactivity component contributed by the sympathetic nervous system. Similarly, ziconotide has also been in trials for several pain indications (Wallace et al., 2008; Vetter and Lewis, 2012; Deer et al., 2019). One consideration for the use of this drug is that ziconotide is sympatholytic when administered intravenously, and produces orthostatic hypotension, which is consistent with its high level of expression in the SCG (Figure 3). We note that the SCG is considered as representative of sympathetic ganglia, but that future studies examining more of the sympathetic chain may also be valuable for refining the predicted drug selectivity along the neuraxis. The theme of using transcriptional signatures to examine potential side effects is further examined throughout the paper.

Of the ganglia examined, the SCG is unique in its comparative homogeneity, which results in a small number of genes expressed at extremely high levels relative to the sensory ganglia. Some of the genes which are highly expressed in the SCG are Neuropeptide Y (*Npy*), Dopamine beta-hydroxylase (*Dbh*), and the vesicular and plasma membrane norepinephrine transporters, *Slc18a2* and *Slc6a2*, respectively (Figures 1A, 9). The SCG also expressed very high levels of TrkA (neurotrophic receptor tyrosine kinase 1, *Ntrk1*), the tyrosine kinase receptor for nerve growth factor (NGF). The extraordinary amount of *Ntrk1* transcript present within these cells may explain their sensitivity to NGF deprivation (Smeyne et al., 1994), and is a potential consideration for the use of chronic anti-NGF antibody-based analgesia. These concerns have been raised in part due to experiments showing that sequestration of NGF in embryonic animals using antibody treatment leads to destruction of sympathetic ganglia (Levi-Montalcini and Booker, 1960). NGF leads to extension of dendrites in adult sympathetic neurons, whereas NGF sequestration by the use of NGF antibodies leads to retraction of dendrites in sympathetic ganglia, and decreased soma size (Ruit et al., 1990; Belanger et al., 2017). These effects on dendrites and soma size reverse upon discontinuation of anti-NGF antibodies (Belanger et al., 2017). Nonetheless one proposed use is for analgesia in osteoarthritis for which treatment can be required for many years, potentially impacting ganglionic neurons, although the high levels of receptor may be able to scavenge trace amounts of NGF and maintain neuronal integrity.

Several other genes play somewhat underappreciated roles in ganglionic functions, yet are brought sharply into prominence by their very high level of expression. An example in the SCG is Cytochrome B561 (*Cyb561*), a transmembrane electron transport protein specific to a subset of secretory vesicles containing catecholamines and amidated peptides (Perin et al., 1988). This enzyme is indirectly involved in norepinephrine and neuropeptide biosynthesis by supplying reducing equivalents to the intravesicular enzymes dopamine-beta-hydroxylase and peptidylglycine alpha-amidating monooxygenase (PAM) (Eipper and Mains, 1988). The latter enzyme amidates the carboxy terminal amino acid of many neuropeptides, including Neuropeptide Y (NPY) which is very highly expressed in the SCG. We have focused on the main catecholaminergic neuronal population in the SCG, as this ganglion contains very high levels of the molecular components necessary for noradrenergic transmission. For example, using immunostaining, nearly every cell contained tyrosine hydroxylase reactivity (Figure 2). Despite this major population, the SCG also contains a subpopulation of cholinergic neurons that innervate sweat glands and erector pili muscles of hair follicles. This subpopulation recently has been subjected to an independent transcriptomic analysis (Furlan et al., 2016).

Whereas the involvement of the SCG in pain is largely indirect, the NDG is a major sensory ganglion involved in the detection of visceral stimuli, and shares many nociceptor molecular markers with the trigeminal and dorsal root ganglia (Figure 3). An example of the “nociceptive character” of the NDG is the high expression of the acid-sensing ion channel, Asic3. This finding is consistent with the data from Sutherland et al. (2001), which found that Asic3 was the primary sensor of myocardial acidity, and consequently, cardiac ischemic pain. The NDG is also remarkable for its high levels of transient receptor potential channels, especially TRPV1 and TRPA1 (Figure 3), where the level of expression is equal to or greater than that of the DRG or trigeminal, and where we observed a higher degree of colocalization between these two ion channel transcripts (Figures 2D–F). While the NDG is not thought to participate in nociception *per se*, it is likely capable of detecting noxious events due to the expression of sensory transducing molecules shared between this ganglion and the DRG. In its role in sensing the physiological status of the gut, the nodose must respond to a broad range of chemical stimuli to identify nutritive (de Lartigue and Diepenbroek, 2016) and potentially problematic stimuli (Yu et al., 2005). The broadly responsive chemosensory functions of TRPA1, for example, are consistent with the broadly responsive character of NDG afferents (Schwartz et al., 2011; Meseguer et al., 2014). In this instance, the NDG and TRPA1 have both been implicated in tussive responses (Birrell et al., 2009; Grace et al., 2012; Patil et al., 2019), which is consistent with the high expression of *Trpa1* in rat ganglia in this study. While not all of the functions of noxious stimulus detection in the nodose have been fully elucidated, it is notable that the nodose projects to the area postrema, which is known for regulating nausea and visceral discomfort (Miller and Leslie, 1994). This is consistent with the idea that visceral pain is often accompanied by nausea and appetite suppression. Additionally, the nodose participates in reflexive responses during gustation and coughing (Taylor-Clark et al., 2009). These responses require similar sensory transduction events as those occurring in the DRG, and could explain the overlap in expression of many of the receptors responsible for its low threshold mechanosensitive properties (Brierley et al., 2012). One of the reflexive responses that the nodose conveys is that of tussive responses, and vagal lesions are known to affect coughing and swallowing (Rontal and Rontal, 1977). Notably, the development of novel antitussive agents has been focused on receptors such as the mu-opioid receptor and P2rx3 which are highly expressed in the NDG, presumably among the sensory neurons that respond to stimuli that evoke cough (Ford, 2012; Trankner et al., 2014; Dicpinigaitis et al., 2020). For example, mu-opioid receptor agonists suppress cough, although these drugs also have other side effects (Bolser, 2010).

Selective regional anesthesia can be administered into a target organ, such as the application of TRPV1 agonists to destroy sensory axons to achieve regional analgesia. It has now been established that the TRPV1+ axon is a sensitive cellular compartment (Sapio et al., 2018), and is destroyed by calcium influx after local TRPV1 agonist application (Caudle et al., 2003; Karai et al., 2004; Neubert et al., 2008). While it has not been fully explored, the high level of TRPV1 in the NDG suggests that these fibers would most likely be lesioned in a similar manner as was demonstrated for the axons originating from DRG and trigeminal nociceptors. The implication of the high degree of overlap in molecular targets between nodose and other sensory ganglia is that peripherally directed analgesics can act at sensory neurons in either the nodose or the DRG afferents when an agent is administered either systemically or into a target organ. The capacity for local administration, therefore, provides a high degree of flexibility and targeting of symptom control. For example, pain and possibly other symptoms from pancreatitis or pancreatic cancer could be treated by local infusion of resiniferatoxin, and will most likely lesion fibers from the NDG in addition to DRG afferents. The effects of lesioning both sets of fibers could be further explored. Another area that has been examined extensively is the ablation of TRPV1+ fibers innervating the lung. For example, these fibers are thought to contribute to pathophysiological inflammation, where resiniferatoxin-induced ablation of these TRPV1 fibers was proposed to reduce such inflammation. Similarly, they have been proposed as one source of pulmonary proinflammatory mediators in severe coronavirus infections in humans (Nahama et al., 2020).

While the nodose and DRG both express a broad set of nociceptive transducing genes such as *Trpv1*, the nodose also contains unique molecular signatures related to satiety that are not found in the DRG. Within the set of nodose enriched satiety genes, we identified the satiety-promoting peptide precursor gene *Cartpt*, and the neuronal “A” paralog of the receptor for cholecystokinin, *Cckar*. Previous studies have demonstrated that vagal afferents stimulated with cholecystokinin peptide produce and release CART peptide, delineating part of a satiety circuit regulated by these neurons (de Lartigue et al., 2007). Of interest, several other known genes coding for satiety-related receptors are highly enriched in the nodose, including the glucagon-like peptide 1 receptor (*Glp1r*), which also suppresses appetite and has been targeted using synthetic analogs for type II diabetes and weight loss (Vilsboll et al., 2012). Also among these genes is the neuropeptide Y receptor type 2 (*Npy2r*) which is a receptor for both NPY and peptide YY (Gerald et al., 1995), variants of which are associated with obesity (Torekov et al., 2006). In this context, it is likely that this receptor responds to the anorexigenic peptide YY secreted from the digestive tract in response to feeding (Holzer et al., 2012). Somatostatin, likewise, is also secreted by various parts of the digestive tract including the duodenum and pancreatic islets, and would signal to nodose neurons through the somatostatin receptor 4 (*Sstr4*) which is highly enriched in this ganglion. Interestingly, Sstr4 has been suggested as an analgesic target (Pan et al., 2008; Van Op den Bosch et al., 2009; Schuelert et al., 2015). The corticotropin-releasing hormone receptor 2 (*Crhr2*) gene is also among the highly enriched nodose receptor genes, and CRH signaling is known to be anorexigenic (Mastorakos and Zapanti, 2004). In summary, many of the highly enriched genes in the nodose code for receptors that can respond to secreted factors from the gut, and which inhibit feeding behaviors, consistent with the role of the vagal afferents in signaling satiety (Berthoud, 2008; Kentish and Page, 2015). Notably, these responses can be indirect in some cases, receiving signals transmitted by enteroendocrine or neuropod cells in the gut lining (Kaelberer et al., 2018). The nodose also has a high degree of enrichment for the ionotropic serotonin receptors *Htr3a* and *Htr3b* (Figure 6 and Supplementary Figure 6), which are the pharmacological targets of antiemetic and anti-nausea drugs such as ondansetron (Grace et al., 2012). These receptors are thought to inhibit vagal signaling to the nucleus of the solitary tract around the area postrema (Babic and Browning, 2014). While drugs targeting vagal 5-HTRs may not directly affect satiety, nausea is potently anorexigenic. These functions of the NDG, in concert with its remarkable level of *Oprm1* expression may serve to partially explain opioid-induced nausea (Smith et al., 2012), especially given the prominent role of the nodose in nutrient sensing and emesis.

Further exploration of the NDG was performed to ascertain more information about the cell types and the combinatorial co-expression patterns of nodose genes. The existing literature suggests many functions of vagal afferents, but is lacking detailed information about the nature of the cell types and the presence of signal-detecting molecules on these cells. In general, these co-staining analyses revealed that most cells exhibit markers of genes associated with satiety, nausea, and monitoring of the gut consistent with the idea that this is a prominent function of vagal afferents. The identification of a capsaicin-responsive, cholecystokinin-responsive population of sensory neurons innervating the peritoneal space and synapsing in the area postrema (Monnikes et al., 1997) is consistent with Trpv1+/Cckar+ vagal afferents being involved in nausea. It also supports the observation that administration of cholecystokinin can cause nausea, anxiety and reduction of appetite (Miaskiewicz et al., 1989; Greenough et al., 1998). From our analyses, there appears to be substantial overlap between nociceptive sensory neurons and neurons that sense and/or transmit satiety signals, suggesting a joint function of some sensory neurons in this ganglion. We also note that the DRGs sampled in the present analyses concentrate on those ganglia having a more uniform representation of somatic primary afferents. DRGs associated with visceral innervation may have a higher degree of overlap with the nodose, and could be more directly comparable with vagal afferents. Future studies may be able to further refine the differences between visceral and somatic nociceptive modalities by including a comparison of visceral DRG afferents as well, through either tracing or sorting, or selective examination of DRGs containing substantial visceral representation.

Nicotinic receptors have an established role in addiction, autonomic physiology and analgesia, making them potentially useful drug targets in several areas of medicine, but also introducing the possibility of dose-limiting side effects. The analgesic role in particular for nicotinic agonist drugs such as epibatidine has been proposed to be mediated by α7 nicotinic receptors in the DRG and dorsal spinal cord (Umana et al., 2013). More recent investigations using RNA-Seq have shown that α7 receptors are not expressed strongly in murine TRPV1+ nociceptive DRG neurons, while other subunits such as the α6 subunit appear to be more promising (Goswami et al., 2014a). The present report confirms that, in addition to being located in the nociceptive neurons of the rat DRG, the α6 subunit is also more specific for sensory afferents relative to other peripheral ganglia (Figure 4). One caveat to this, is the expression of α6-containing pentameric configurations in the retina, although it is unclear how much of this configuration is present relative to DRG, for example. The α7 homomer can be found in modulatory interneurons within the dorsal horn, as well as throughout the brain, potentially modulating pain sensitivity in the CNS. By contrast, the α6β2β3 pentamer is thought to be more specific for sensory ganglia (Genzen et al., 2001; Dussor et al., 2003). While expression patterns could be explored further, the α6-containing nicotinic receptors do not appear to be strongly detected in grossly dissected brain regions, which may suggest the capability for enhanced pharmacological targeting to peripheral ganglia (Consortium, 2013; Uhlen et al., 2015).

The data presented here highlight the idea that RNA-Seq can play a central role in the process of discovery, in the refinement of *in vitro* and *in vivo* physiological experimentation, and to the success of drug development efforts. The fundamental science of ganglionic transcriptomics is evolving through comparative approaches which now includes mouse, rat, dog and human (Iadarola et al., 2018; Ray et al., 2018; Sapio et al., 2018). These efforts are also being amplified further using single cell sequencing, cell sorting, and microscopy to elucidate cell populations in the nodose and jugular ganglia (Wang et al., 2017; Kupari et al., 2019; Kaelberer et al., 2020). These single-cell investigations may be particularly useful for clarifying neural and non-neural cell type differences between the ganglia, and could be useful in identifying differences in glia and immune cell compositions in particular. However, much more needs to be done to really understand how the external world communicates with the body, and the body communicates with itself. Many parasympathetic ganglia are embedded in the end organ itself or in a bony fossa and are difficult to locate or extricate. Those of the head and neck perform crucial functions in controlling tears, cough, nasal blood flow, parotid gland secretion and pupillary dilation. Obtaining these from the human at autopsy also depends on donation restrictions. Nonetheless, medical disorders are attributable to all of these ganglia and establishing foundational data based on next-generation sequencing is a necessary goal for advancing our knowledge of the range of peripheral nervous system functions.

## Data Availability Statement

The datasets presented in this study can be found in online repositories. The names of the repository/repositories and accession number(s) can be found in the article/Supplementary Material.

## Ethics Statement

The animal study was reviewed and approved by the Animal Care and Use Committee at the National Institutes of Health.

## Author Contributions

SI and MI conceptualized the project. MS and MI wrote the manuscript. MS, FV, AL, DM, JK, HP, SI, MI, and AM edited the manuscript. MS, FV, AL, DM, JK, DL, HP, and VL performed the experiments. MS, FV, and AL constructed the visualizations. MS, FV, AL, and MI performed the analysis. SI, MI, and AM acquired the funding. SI, MI, and AM supervised the project. All the authors contributed to the article and approved the submitted version.

## Conflict of Interest

The authors declare that the research was conducted in the absence of any commercial or financial relationships that could be construed as a potential conflict of interest.
