# Supplementary material for: Comparative Analysis of Dorsal Root, Nodose and Sympathetic Ganglia for the Development of New Analgesics
Source: Front Neurosci. 2020 Dec 23;14:615362. doi: 10.3389/fnins.2020.615362 (PMC7793666; doi:10.3389/fnins.2020.615362)
Supplement: Supplementary file 1 [file Data_Sheet_1.docx]

Comparative analysis of dorsal root, nodose and sympathetic ganglia for the development of new analgesics.

**Matthew R. Sapio^1^, Fernando A. Vazquez^1^, Amelia J. Loydpierson^1^, Dragan Maric^3^, Jenny J. Kim^1^, Danielle M. LaPaglia^1^, Henry L. Puhl^2^, Van B. Lu^2^, Stephen R. Ikeda^2^, Andrew J. Mannes^1^, Michael J. Iadarola^1*^**

^1^Section on Anesthesia, National Institutes of Health, Clinical Center, Department of Perioperative Medicine, Bethesda, MD, USA

^2^Section on Neurotransmitter Signaling, National Institute on Alcohol Abuse and Alcoholism, National Institutes of Health, Bethesda, MD, USA

^3^Flow and Imaging Cytometry Core Facility, National Institute of Neurological Disorders and Stroke, National Institutes of Health, Bethesda, MD, USA

***Correspondence:**Michael J Iadarola, Ph.D.
Department of Perioperative Medicine
Clinical Center
Building 10, Room 3D56
10 Center Drive, MSC 1510
National Institutes of Health
Bethesda, MD 20892-1510
Office: 301-496-2758
[michael.iadarola@nih.gov](mailto:miadarol@cc.nih.gov)

**Associated Sequence Read Archive BioProjects:**

**PRJNA681229** Comparative analysis of dorsal root, nodose and sympathetic ganglia (Rattus norvegicus)

**PRJNA313202** DRG vs Sciatic Nerve transcriptomics

Keywords: Opioid, Nodose, DRG, ganglion, RNA-Seq. (Min.5-Max. 8)

| **Gene** | **Catalogue#** |
| --- | --- |
| *Trpv1* | 501161 |
| *Trpa1* | 312511 |
| *Glp1r* | 315221 |
| *Cartpt* | 449511 |
| *Cckar* | 412091 |
| *Sstr4* | 544061 |
| *Gpr160* | 515121 |
| *Htr3a* | 480041 |
| *Htr3b* | 469311 |
| *Npy2r* | 414481 |
| *P2rx3* | 543401 |
| *Oprm1* | 410691 |

Supplementary Table 1. Catalogue numbers of RNAScope probes used in multiplex fluorescent *in situ* hybridization experiments. All probes are currently available for purchase in the Advanced Cell Diagnostics catalogue. None of these probes were specifically designed to be splice-variant specific, and were designed against the major transcript in *Rattus* *norvegicus*.

| **Panel** | **Cells** | **Plotted** | **N** | **520** | **570** | **620** | **690** |
| --- | --- | --- | --- | --- | --- | --- | --- |
| A | 352 | 345 | 3 | *Cartpt* | *Sstr4* | *Cckar* | *Glp1r* |
| B | 342 | 248 | 3 | *Cartpt* | *Glp1r* | *Trpa1* | *Trpv1* |
| C | 706 | 702 | 3 | *Htr3b* | *Glp1r* | *Trpv1* | *Htr3a* |
| D | 295 | 268 | 3 | *Trpv1* | *Glp1r* | *Cckar* | *Npy2r* |
| E | 234 | 215 | 3 | *Cartpt* | *Oprm1* | *Trpv1* | *P2rx3* |
| F | 293 | 189 | 3 | *Htr3b* | *Oprm1* | *Glp1r* | *Trpv1* |

Supplementary Table 2. Number of cells counted. For the counts of probe-positive cells in Figure 6, 7 total 4-plex combinations were performed. This table shows the number of cells counted in each combination (Cells), as well as how many of these counted cells are shown in the graphs in Figure 6 (Plotted). Note that combinations with less than 10 total counts were not plotted as they may not represent a substantial or reproducible population of ganglionic neurons. N=3 ganglia (each from a different animal) were considered in this analysis.

**
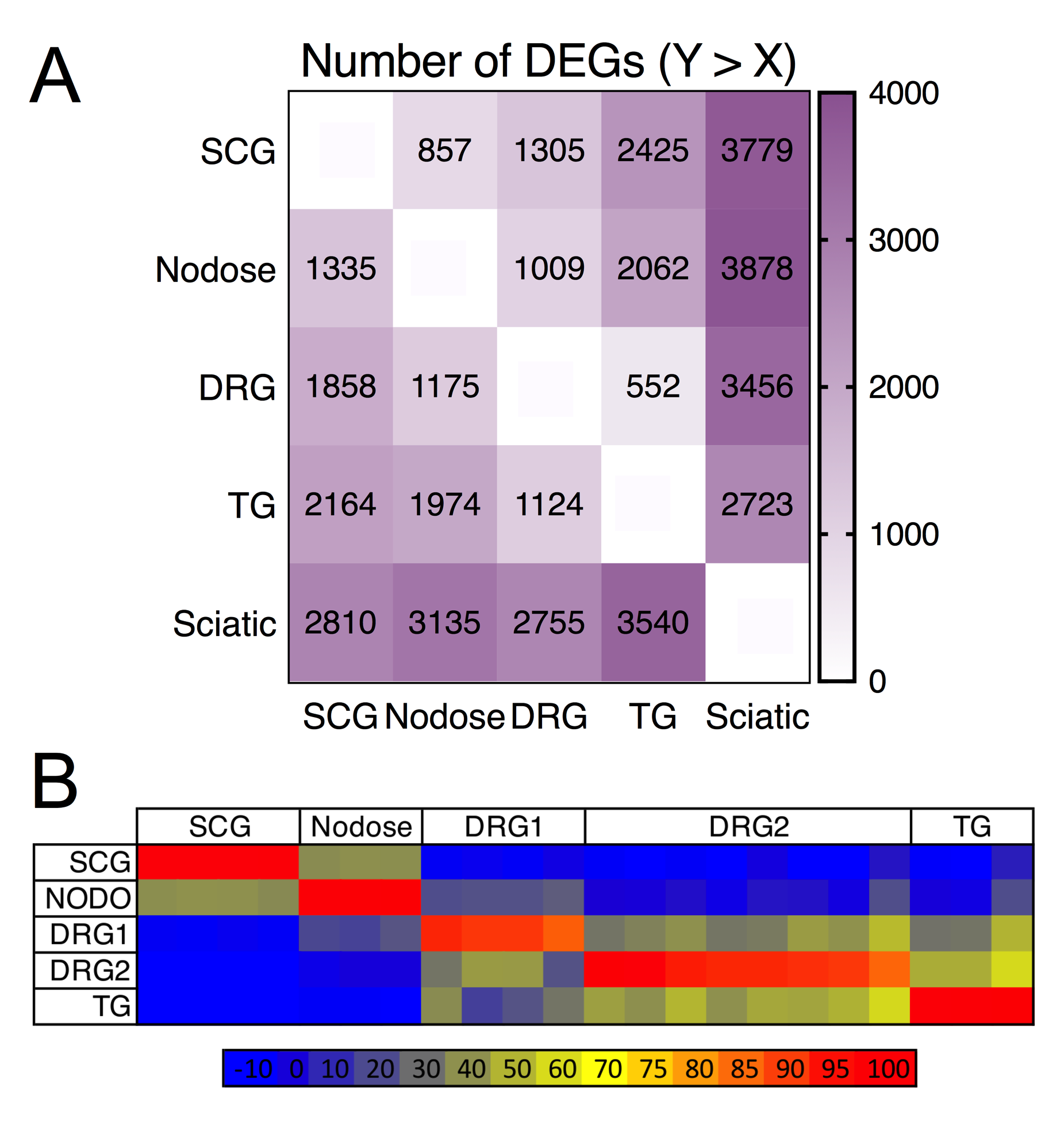
**

**Supplementary Figure 1. Quality control and statistical summaries of ganglionic RNA-Seq datasets. A.** The number of differentially expressed genes calculated in MAGIC (LaPaglia et al., 2018) was plotted in a matrix where each ganglionic dataset was compared to one another. More similar datasets such as the DRG and trigeminal had fewer differentially expressed genes, while the non-neural sciatic nerve was in general the most differential compared to the neural tissues. A small number of siginificant genes in the sciatic nerve come from adherent adipose tissue not anatomically proximal to ganglionic tissue (Sapio et al., 2016b). **B.** A correlation matrix was plotted to compare the overall similarity of expression of all genes expressed above 4 sFPKM in each of the ganglionic datasets. This includes two batches of DRGs collected at different times. The 8 DRGs in batch 2 were published previously(Sapio et al., 2016b). In general the corrrelation among samples in the same group was high. A single nodose sample was excluded based on lack of correlation with any other sample, most likely due to tissue degradation before sequencing, or sequencing error.


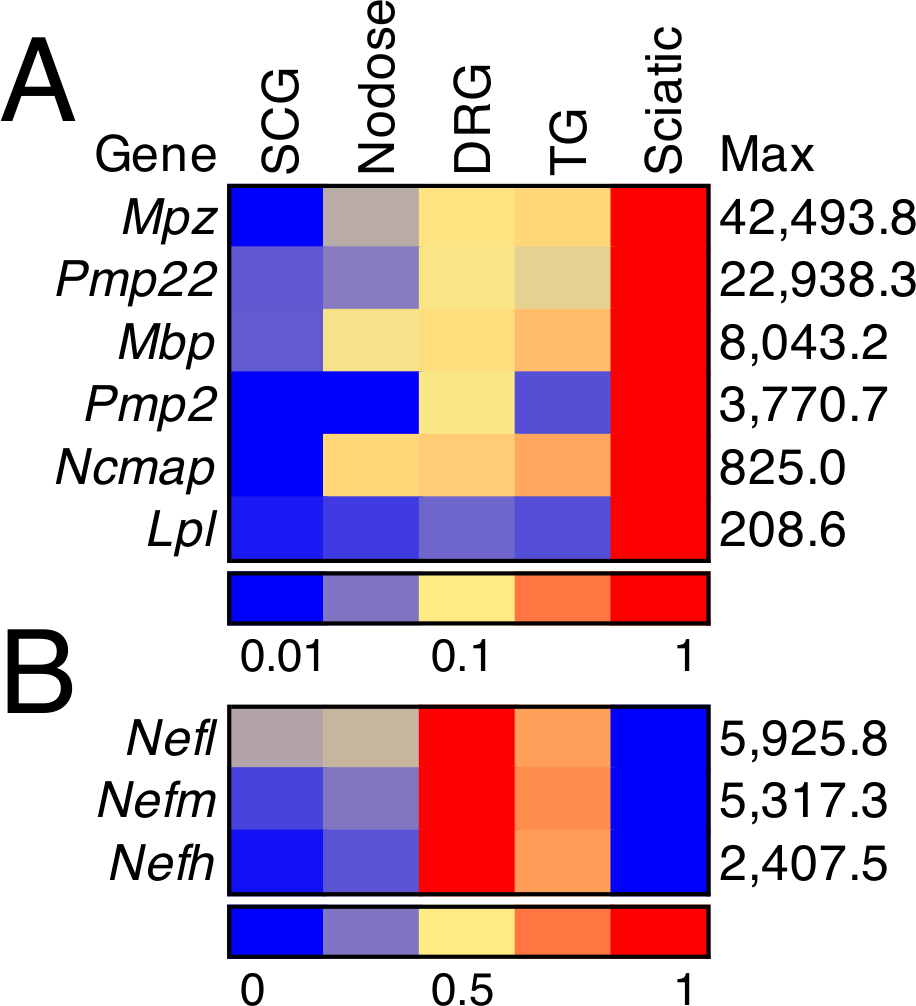


**Supplementary Figure 2. Mapping of myelin marker genes in peripheral ganglionic RNA-Seq datasets.** The sciatic nerve was included in these comparisons to provide a marker of non-neuronal cell populations, most notably peripheral myelinating Schwann cells, which are present in all of the ganglia observed. The large degree of difference in gene expression between samples is expressed using log10 scale, with sciatic nerve showing extremely high (>40,000 sFPKM) expression of the most highly expressed peripheral myelin protein, *Mpz*. The Nodose, DRG and Trigeminal ganglia have comparable amounts of myelinated fibers, with higher amounts in the DRG and TG than in the nodose. The SCG has by far the least amount of peripheral myelination, consistent with the low number of myelinated fibers in this ganglion. The nodose consists of roughly 20% myelinated A-fibers, with the remainder as unmyelinated C-fibers(Li et al., 2008).

**
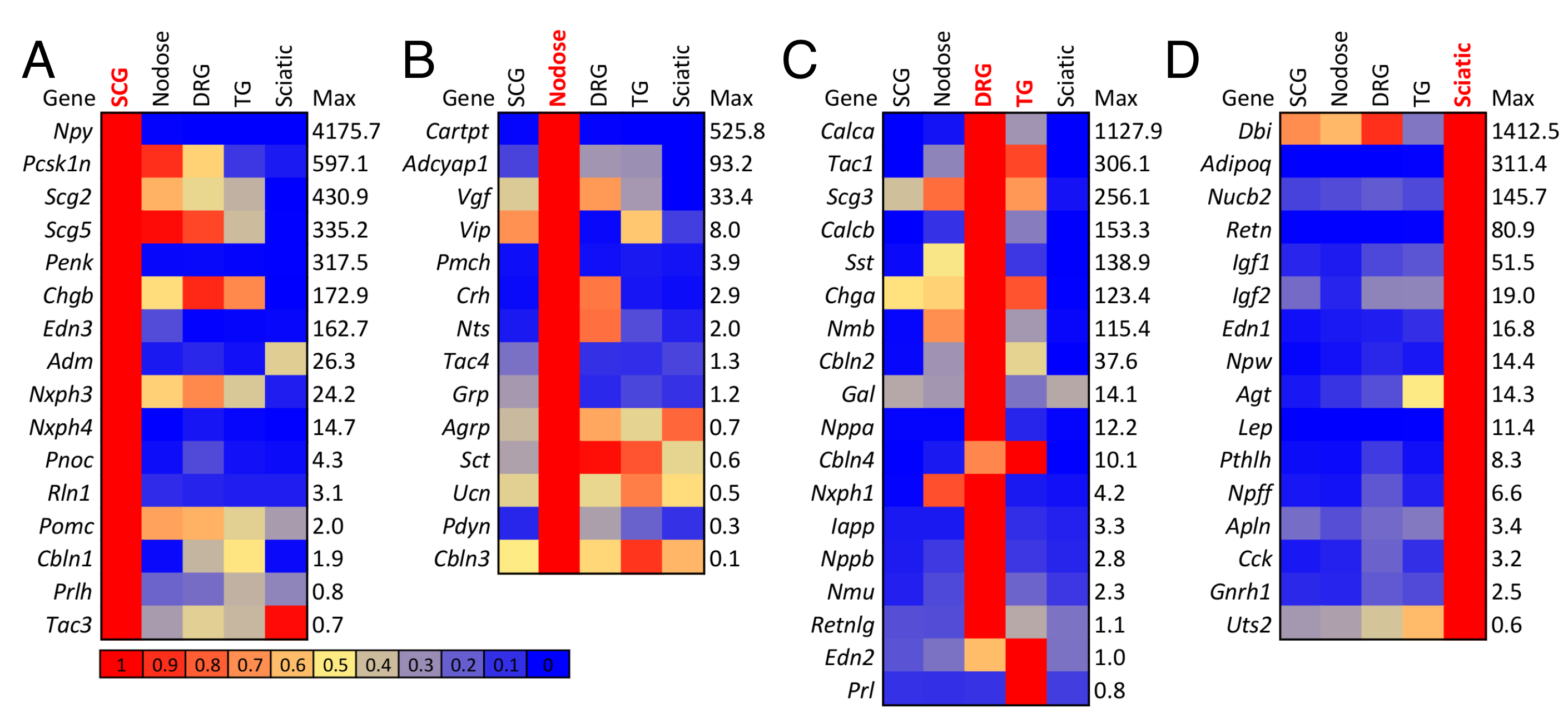
**

**Supplementary Figure 3. Peptide transmitter precursor genes enriched in peripheral ganglia and peripheral nerve.** Genes were separated by functional category according to IUPHAR data accessed at https://www.guidetopharmacology.org/helpPage.jsp. Peptide precursor genes were shown for all ganglia examined in the present study. Each list is sorted by maximum expression (sFPKM) in any one sample. **A.** The highest expressed genes in SCG. **B.** The highest expressed genes in nodose. **C.** The highest expressed genes in DRG/trigeminal. **D.** The highest expressed genes in sciatic nerve. In all plots, heatmaps were constructed as described elsewhere in the manuscript. For each gene, expression was normalized by row (maximum value set to 1), and colored according to the flame scale. The maximum value in sFPKM is also shown in the column marked Max. The methodology used to produce the graphs in this figure are identical to those in in Supplementary Figures 4-7 except where noted.


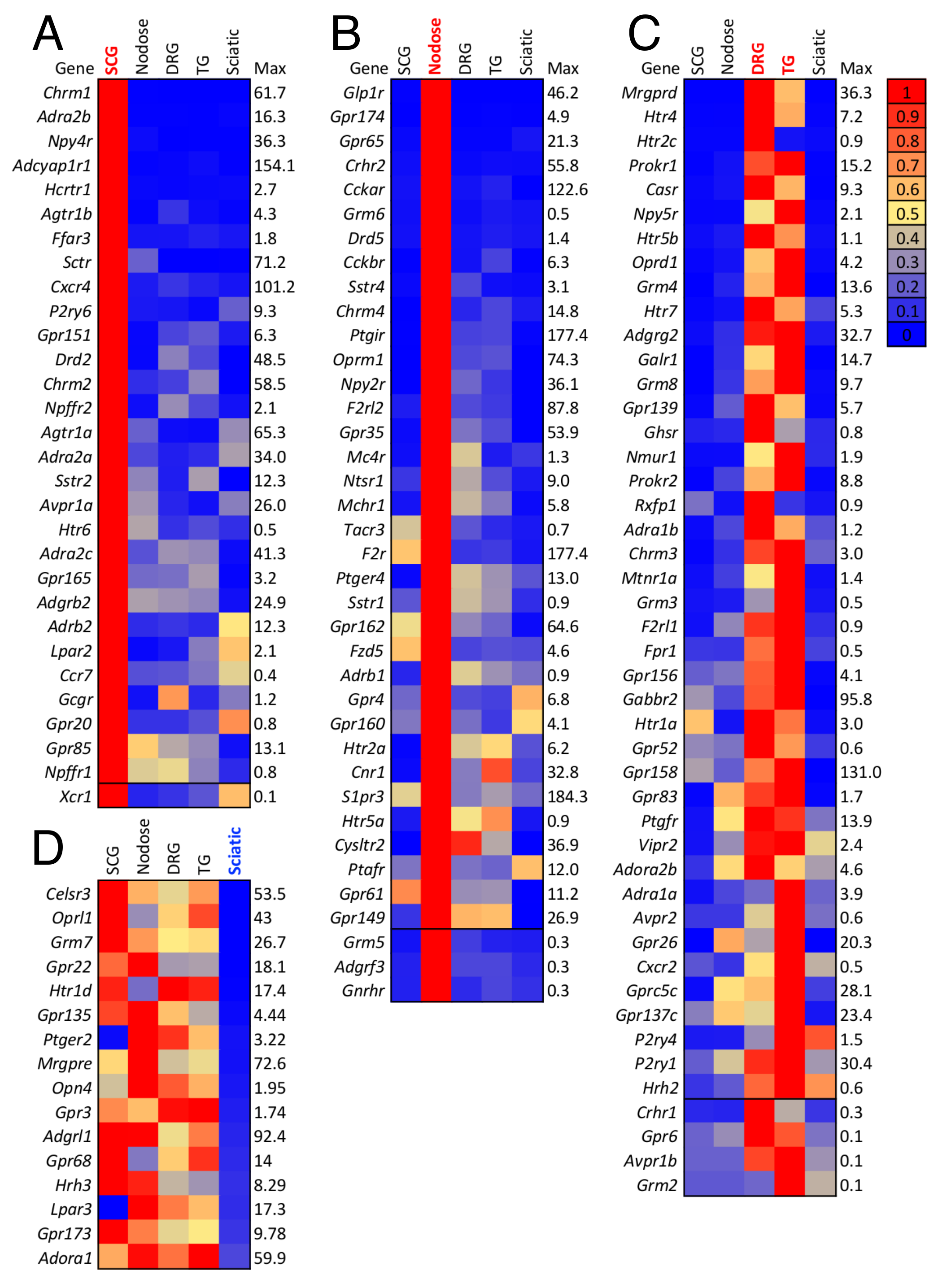


**Supplementary Figure 4. G Protein-coupled receptors enriched in each peripheral ganglia.** GPCR genes were compared for level of expression between the five datasets. Genes enriched in SCG (A), Nodose (B) are plotted if the sFPKM value in those datasets exceeds 3x the average of the expression value in the other 4. For the DRG and TG map, genes are plotted if the sFPKM value exceeds 3x the value of the other 3 for either the DRG or the TG. Genes which are not in the other plots, but which are 5-fold enriched in the average of the 4 ganglionic datasets relative to the sciatic nerve are shown in (D). Genes with a maximum expression in any tissue examined of 0.3 sFPKM or less are shown at the bottom of each chart.


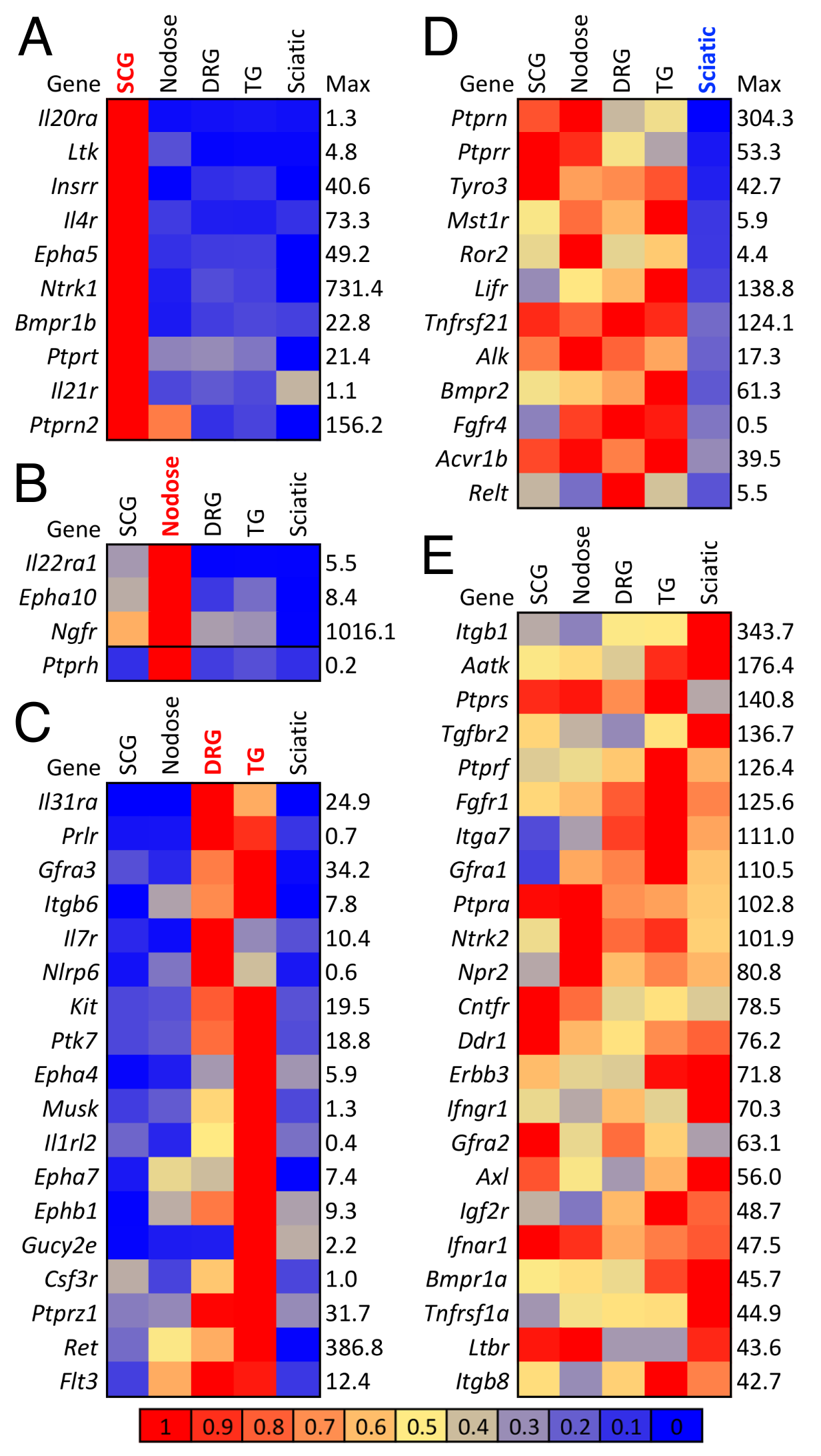


**Supplementary Figure 5. Catalytic receptor genes enriched in peripheral ganglia.** Catalytic receptor genes were shown for all ganglia examined in the present study. Each list is sorted by maximum enrichment in each ganglion. **A.** The most highly enriched genes in SCG. **B.** The most highly enriched genes in nodose. **C.** The most highly enriched genes in DRG/trigeminal. **D.** The highly enriched genes in all four ganglia relative to sciatic nerve. **E.** The most highly expressed genes with no little/no enrichment across the 5 tissues examined.


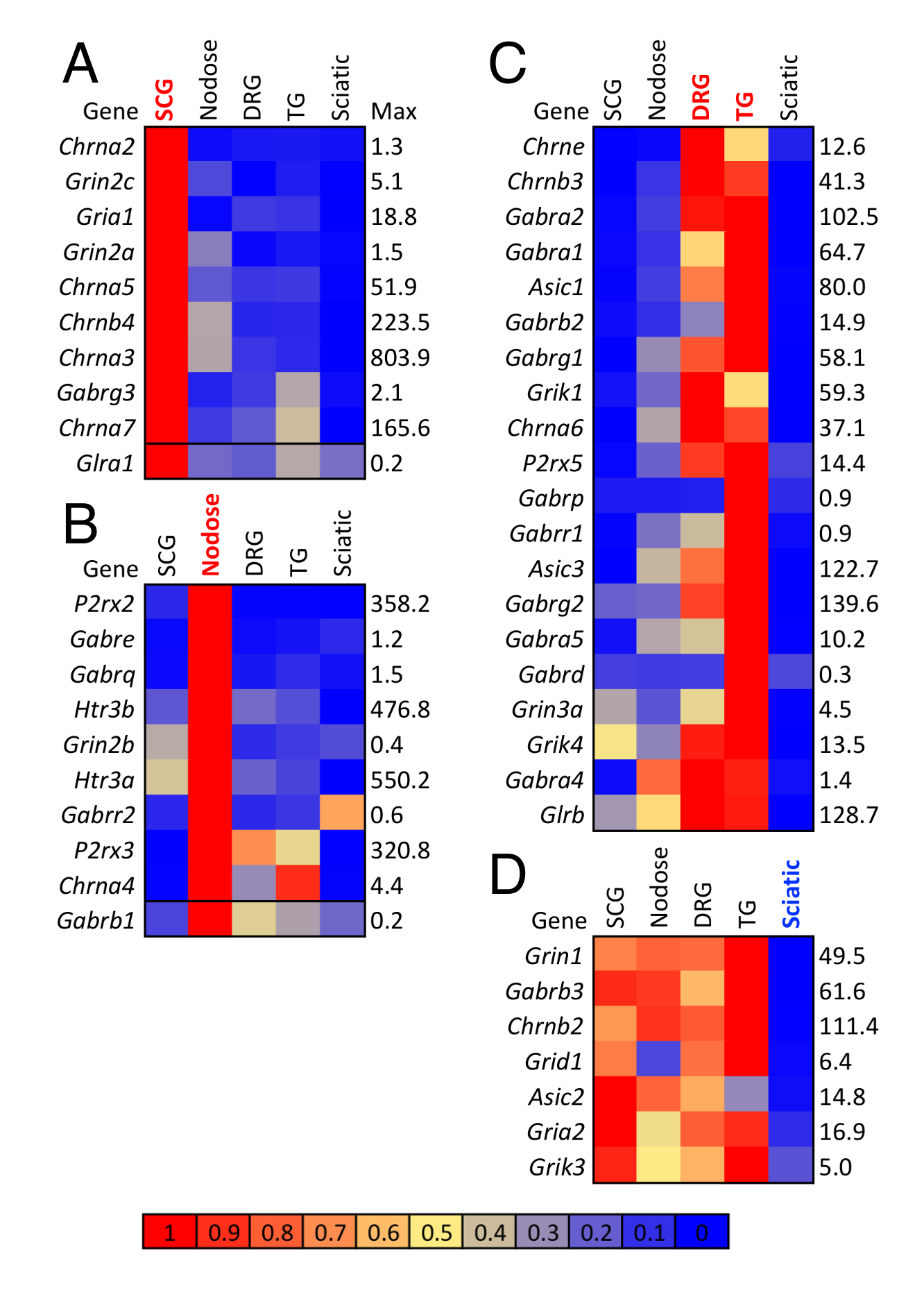


**Supplementary Figure 6. Ligand-gated ion channel genes enriched in peripheral ganglia.** Ligand-gated ion channel genes were shown for all ganglia examined in the present study. Each list is sorted by maximum enrichment in each ganglion. **A.** The most highly enriched genes in SCG. **B.** The most highly enriched genes in nodose. **C.** The most highly enriched genes in DRG/trigeminal. **D.** The highly enriched genes in all four ganglia relative to sciatic nerve.


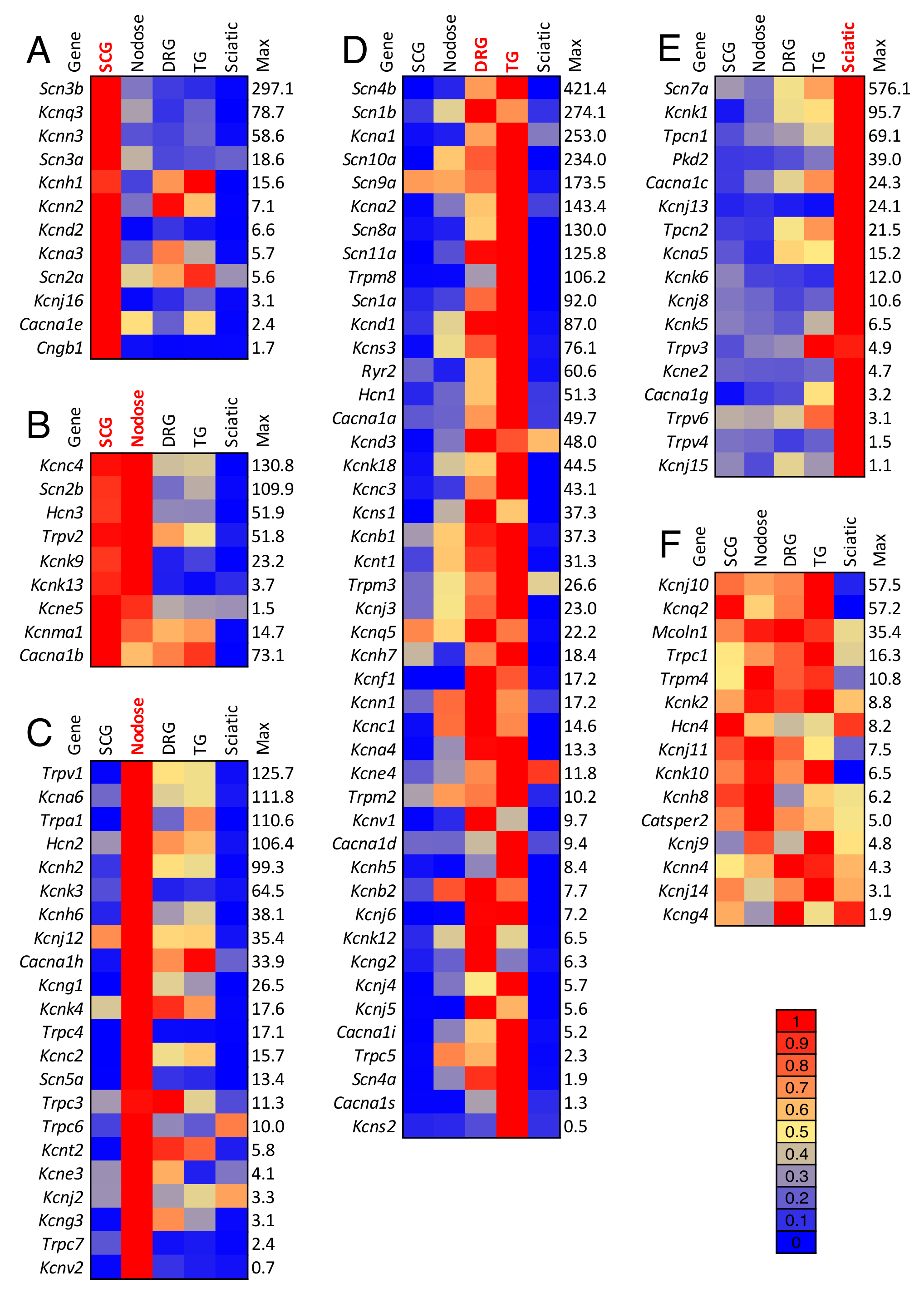


**Supplementary Figure 7. Voltage-gated ion channels.** Voltage-gated ion channel genes were shown for all ganglia examined in the present study. Each list is sorted by maximum expression (sFPKM) in any one sample. **A.** The highest expressed genes in SCG. **B.** The highest expressed genes enriched in SCG and nodose relative to the other three tissues. **C.** The highest expressed genes in nodose. **D.** The highest expressed genes in DRG/trigeminal. **E.** The highest expressed genes in sciatic nerve. **F.** The most highly enriched genes with little/no enrichment across the 5 tissues examined. Note that some genes in this category are enriched relative to the sciatic nerve.
